# Macrocyclic Molecular Glues for the 14‐3‐3/ChREBP Interaction: Affinity and Cooperativity in an Inverse Relationship

**DOI:** 10.1002/anie.202521678

**Published:** 2025-12-18

**Authors:** Marloes A. M. Pennings, Merel A. W. van den Bosch, Ansgar Oberheide, Carlo J. A. Verhoef, Christian Ottmann, Albert J. Markvoort, Galen P. Miley, Luc Brunsveld

**Affiliations:** ^1^ Laboratory of Chemical Biology Department of Biomedical Engineering and Institute for Complex Molecular Systems (ICMS) Eindhoven University of Technology Eindhoven 5600MB The Netherlands; ^2^ Synthetic Biology Group Department of Biomedical Engineering and Institute for Complex Molecular Systems (ICMS) Eindhoven University of Technology Eindhoven 5600MB The Netherlands

**Keywords:** Cooperativity, Macrocycle, Molecular glue, Protein–protein interactions, Structural biology

## Abstract

Molecular glues (MGs) stabilize protein–protein interactions (PPIs) by simultaneously binding two or more proteins at their composite interface. Macrocycles present attractive properties as MGs, including large contact surfaces to address the often flat and undefined composite PPI interfaces, but their structure‐based design has remained intangible. We have designed peptidomimetic macrocycles capable of enhancing the PPI between 14‐3‐3 and the carbohydrate response element binding protein (ChREBP), a regulatory transcription factor. Biophysical characterization of these MGs revealed the importance of optimized linker length, displaying a reduced entropic cost compared to the linear counterparts, while preserving key contacts with 14‐3‐3. Binding assays demonstrated that the macrocycles selectively and cooperatively stabilized the 14‐3‐3/ChREBP complex, with an intriguing inverse relationship between intrinsic binding affinity to 14‐3‐3 and cooperativity in PPI stabilization. Ternary co‐crystal structures of the macrocycles binding at the composite 14‐3‐3/ChREBP interface provided a molecular rationale for the affinity and cooperativity differences. Overall, this study highlights structural, kinetic, and thermodynamic features that guide effective macrocyclic MG design and brings forward the crucial interplay of affinity and cooperativity in stabilizing PPIs.

## Graphical abstract

Macrocycles are effective molecular glues to stabilize the 14‐3‐3/ChREBP interaction. Linker length modulation uncovered an inverse correlation between the macrocycle binding affinity to 14‐3‐3 and its cooperativity in 14‐3‐3/ChREBP stabilization. Co‐crystal structures of these macrocycles in the composite 14‐3‐3/ChREBP binding pocket revealed structural clues behind this affinity and cooperativity interplay.

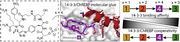

Protein–protein interaction (PPI) modulation, especially via molecular glues (MGs) that bind at the PPI interface, is an entry to target proteins not typically amenable via classical drug discovery.^[^ ^1^, ^2^ ^]^ While small molecules are an important therapeutic class, they often have limited surface area compared to the typically large and flat interfaces of PPIs, making it challenging to design potent and selective small molecule modulators for PPIs that lack well‐defined pockets.^[^ ^3^, ^4^ ^]^ Inversely, peptides can offer a larger contact surface area and improved selectivity, yet their flexibility can lead to unfavorable binding energies and kinetics or to pharmacokinetic challenges. Macrocyclization helps to overcome such drawbacks by constraining the peptide into its bioactive conformation, improving its pharmacokinetic parameters and often increasing potency through reduction of entropic penalties.^[^ ^5^ ^]^

Natural macrocyclic peptides are used as therapeutics and inspired the design of synthetic derivatives with improved potency and selectivity.^[^ ^6^, ^7^ ^]^ Moreover, technological advances now allow for the de novo design of macrocycles against targets of interest.^[^ ^5^, ^8^ ^]^ Complementing these methods, deep learning strategies have further unlocked the computational design of large macrocyclic libraries, as illustrated by potent and selective inhibitors of enzymes and receptors.^[^ ^9^, ^10^ ^]^ PPI inhibition using macrocyclic peptides has been successfully applied, for PPIs such as YAP/TEAD, β‐catenin/TCF, and MLL/KIX.^[^ ^11^, ^12^, ^13^, ^14^ ^]^ Interestingly, the first identified PPI stabilizers, or MGs, were also natural product macrocycles; Rapamycin and Cyclosporin A.^[^ ^15^ ^]^ Notwithstanding, despite its enormous potential for drug development and conceptual understanding of PPI stabilization, the structure‐based design of de novo synthetic macrocycles as MGs remains underexplored.^[^ ^16^, ^17^, ^18^ ^]^

Within the many protein complexes of therapeutic relevance, 14‐3‐3 scaffold proteins are an interesting target due to their extensive interactome. These PPIs have broad consequences affecting client protein localization, activity or further complex formation.^[^ ^19^ ^]^ A unique client protein of 14‐3‐3 is the carbohydrate response element binding protein (ChREBP), which interacts via an α‐helix, independent of phosphorylation.^[^ ^20^ ^]^ Binding of 14‐3‐3 to this glucose responsive transcription factor inhibits its nuclear localization and transcriptional activity. Small molecule MGs of the 14‐3‐3/ChREBP complex have been shown to rescue β‐cells in glucolipotoxic conditions.^[^ ^21^ ^]^ Additionally, a nonnatural linear peptide (**pepD**) has been identified as MG of the 14‐3‐3/ChREBP complex.^[^ ^22^ ^]^ Here, we utilized the 14‐3‐3/ChREBP complex as attractive model system to explore the potential and unique characteristics of macrocyclic peptides as MGs. By linker length‐variable cyclization (Figure 1a), we tune the affinity and cooperativity of the macrocycles for the 14‐3‐3/ChREBP complex, resulting in potent macrocyclic MGs for this PPI.

**Figure 1:**
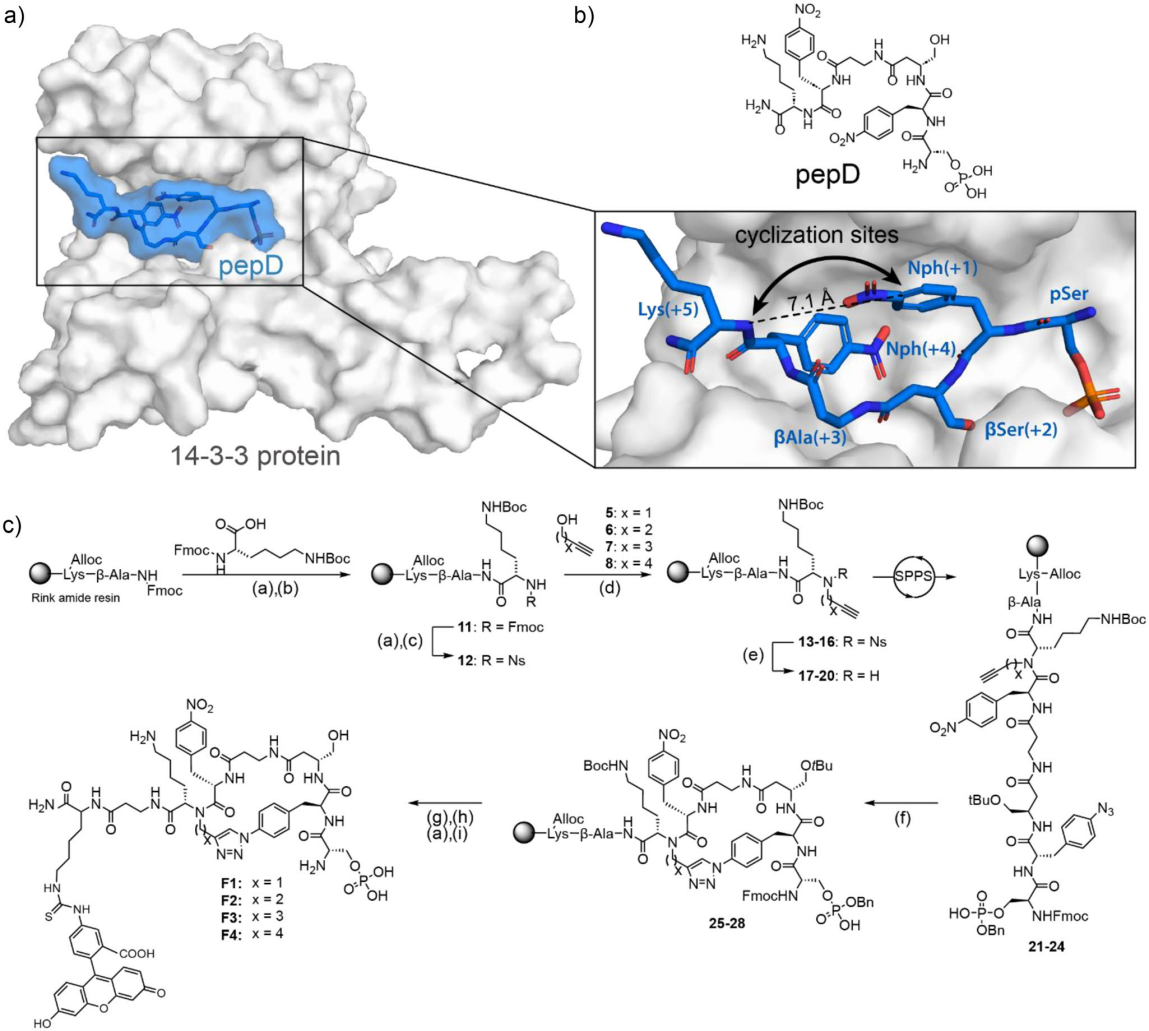
a) Crystal structure of **pepD** (sticks and surface, blue) binding in the pocket of the 14‐3‐3 protein (surface, grey) (PDB 7ZMU). b) Chemical structure of **pepD** and sites selected for the cyclization of **pepD**. c) Synthesis route for macrocycles **F1** (*x* = 1), **F2** (*x* = 2), **F3** (*x* = 3), and **F4** (*x* = 4). Reagents and conditions: (a) 20% piperidine in DMF, rt, 2 × 5min. (b) Fmoc‐AA, HCTU, DIPEA, DMF, rt, 2 h. (c) 2‐Nosyl chloride, 2,4,6‐collidine, DCM, rt, 16 h. (d) Alcohol **5**–**8**, PPh_3_, DEAD, THF, rt, 6 h. (e) β‐mercaptoethanol, DBU, DCM, rt, 1 h. (f) Cu(MeCN)_4_PF_6_, TBTA, DMF, rt, 16 h. (g) Tetrakis (triphenylphosphine)palladium (0), phenylsilane, DCM, rt, 2 × 45 min. (h) Fluorescein isothiocyanate, DIPEA, DMF, rt, 16 h. (i) 95% TFA, 2.5% Tris, 2.5% H_2_O, rt, 2 h.

The crystal structure of **pepD** in complex with 14‐3‐3 was used as a basis for the design of the macrocyclization strategy.^[^ ^22^ ^]^ Two positions, 4‐nitrophenylalanine (Nph(+1), counting from pSer) and the peptide backbone amide nitrogen atom between Lys(+5) and Nph(+4) were chosen as suitable cyclization sites to constrain the peptide in its active conformation (Figure 1b). These cyclization sites were chosen based on their spatial proximity, the lack of hydrogen‐bond involvement of the amide NH, and prior findings that the nitro group of Nph(+4) is not essential for 14‐3‐3 binding affinity.^[^ ^22^ ^]^ A solid‐phase peptide synthesis (SPPS) route was designed to cyclize the Lys(+5) backbone to azido phenylalanine, replacing Nph(+1), by copper‐catalyzed azide‐alkyne cycloaddition (Figure 1c). In preparation for cyclization, an alkyne was installed on the peptide backbone via a nosyl activated Mitsunobu reaction.^[^ ^23^ ^]^ Varying alkyl chain length in the starting alcohols led to precursors for the desired macrocycle sizes (**13**–**16**, *x* = 1–4). Nosyl deprotection and subsequent SPPS with the requisite, nonnatural, amino acids allowed access to the linear scaffolds (**21**–**24**) for CuAAC cyclization (**25**–**28**). The side‐chain of the first lysine residue was orthogonally Alloc deprotected and coupled to fluorescein isothiocyanate (FITC). Subsequent global deprotection and cleavage from the resin afforded fluorescently labeled macrocycles **F1**–**F4**. Non‐labeled macrocycles were synthesized without the first lysine and β‐alanine residues to obtain **2**–**4**.

The binding of macrocycles **F** **1**–**F** **4** to the 14‐3‐3 protein was evaluated using Fluorescence Anisotropy (FA) assays, where 14‐3‐3 was titrated against a constant concentration of **F1**–**F4** (Figure 2a). The shortest macrocycle, **F1**, exhibited the weakest binding affinity, resulting in a partial binding curve (*K*_D_ = 57 ± 16 µM). Macrocycle **F2** showed moderate affinity (*K*_D_ = 5.7 ± 2.1 µM), while macrocycle **F3** had the strongest interaction (*K*_D_ = 0.23 ± 0.06 µM). The longer macrocycle **F4** bound 14‐3‐3 with a lower affinity (*K*_D_ = 2.1 ± 0.8 µM) compared to **F3**, but with a similar affinity as **pepD** (*K*_D_ = 3.6 ± 1.3 µM). A linker length of *x* = 3 thus maximizes affinity to 14‐3‐3 for these macrocycles (Figure S1). The affinity of macrocycles **3**, **4,** and **pepD** for 14‐3‐3 was confirmed by isothermal calorimetry (ITC) and surface plasmon resonance (SPR). Across all assays, the *K*_D_ values were consistent and binding trends among macrocycles aligned, demonstrating the robustness of the measured *K*_D_ values (Figure S1).

**Figure 2:**
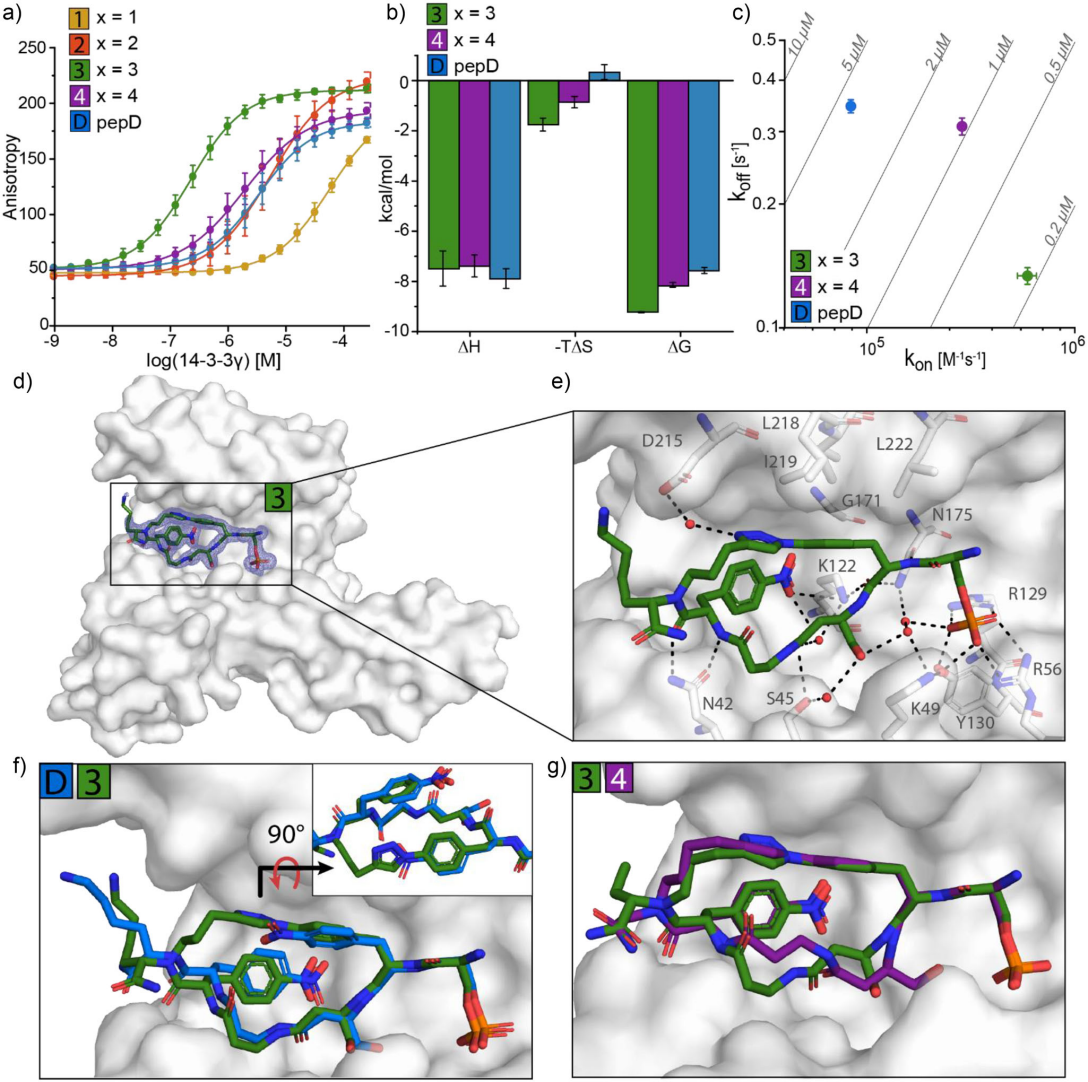
a) Fluorescence Anisotropy assay protein titration of 14‐3‐3γ to FITC‐labeled macrocycles **1**–**4** and **pepD** (10 nM) (mean ± SD, *n* = 3). b) Bar plot of enthalpy (Δ*H*), entropy (−*T*Δ*S*) and Gibbs free energy (Δ*G*) parameters determined by isothermal calorimetry, in which macrocycles **3** and **4** and **pepD** (200 µM) were titrated to 14‐3‐3γ (20 µM) (mean ± SD, *n* = 3). c) Kinetic map showing the relation between association rate (*k*_on_) and dissociation rate (*k*_off_) of peptide binding to 14‐3‐3γ, as determined by surface plasmon resonance. Each dot represents an individual peptide (mean ± SD, *n* = 2). d) Crystal structure of the 14‐3‐3σ protein (grey surface) bound to macrocycle **3** (green sticks). Final 2*F*_o_−*F*_c_ electron density contoured at 1.0*σ*. e) Interactions of macrocycle **3** (green sticks) with 14‐3‐3σ (grey, relevant side chains are displayed as sticks) (polar contacts are shown as black dashed lines). f) Crystallographic overlay of **3** (green sticks) and **pepD** (blue sticks) binding to 14‐3‐3σ (grey surface). g) Crystallographic overlay of macrocycles **3** (green sticks) and **4** (purple sticks) binding to 14‐3‐3σ (grey surface).

Mechanistic ITC studies revealed that the enthalpic contributions (Δ*H*) were consistent across the two studied macrocycles and linear peptide (−7.4 to −7.9 kcal mol^−1^), suggesting that cyclization preserves key interactions between the macrocycles and 14‐3‐3 (Figures 2b and S2; Table S1). Interestingly, the entropic contribution (*T*Δ*S*) was most favorable for the shorter macrocycle **3** (−1.76 kcal mol^−1^), followed by macrocycle **4** (−0.86 kcal mol^−1^), while **pepD** showed a slightly unfavorable entropic contribution (0.33 kcal mol^−1^). The reduced entropic penalty of binding, due to cyclization, made the overall free energy of binding (Δ*G*) become increasingly favorable from **pepD** to macrocycles **4** and **3** (Figure 2b).

Kinetics studies via SPR showed that macrocycles **3** and **4** exhibited significantly higher association rates (*k*_on_), with a 7.6‐ and 3.5‐fold increase compared to **pepD**, respectively (Figures 2c and S3; Table S2). This enhanced *k*_on_ indicates that the conformational changes required for 14‐3‐3 binding are reduced upon cyclization, which aligns with the favorable entropic contribution observed via ITC.^[^ ^24^ ^]^ Notably, macrocycle **3** also displayed a slower dissociation rate (*k*_off_), despite comparable enthalpic contributions across the peptides. This reduced *k*_off_ may reflect a higher activation energy barrier for dissociation, likely due to restricted conformational flexibility in the bound state. Combined, the increased association and decreased dissociation rates significantly enhance the 14‐3‐3 binding affinity of the macrocycles.

Co‐crystal structures of the 14‐3‐3σ/macrocycle complexes were solved to elucidate the molecular mechanism of binding. Clear electron density of macrocycles **2**, **3**, and **4** was observed in the 14‐3‐3 pocket, showing the expected position of the phosphate group interacting with the basic residues R56, R129, and hydrogen bond donor residue Y130 of 14‐3‐3σ (Figure 2d,e; Figures S4 and S5). Hydrogen bonds were observed between the *p*‐nitro group of Nph(+4) and K122, as well as the triazole group and D215 of macrocycle **3** and 14‐3‐3, respectively (Figure 2e). The macrocycle peptide backbone engaged in direct and water‐mediated hydrogen bonding with 14‐3‐3σ residues N42, S45, and N175, likely stabilizing its interaction with the protein. Hydrophobic‐π interactions were observed between the phenyl ring of **3** and the hydrophobic roof of the 14‐3‐3 binding pocket formed by L218, I219, and L222 (Figure 2e).

A crystallographic overlay of macrocycle **3** and **pepD** (Figure 2f) reveals almost identical structural overlap, except for the more flexible exocyclic Lys(+5). The similarity of interactions of macrocycle **3** and **pepD** with 14‐3‐3 aligns with their comparable enthalpic contribution to 14‐3‐3 binding (Figure 2b). Moreover, the overlays of macrocycles **2**, **3**, and **4** similarly reveal a largely consistent binding mode, supporting the enthalpic data, with some deviations in the β‐Ala(+3) and β‐homoSer(+2) residues of macrocycle **4** (Figures 2g and S5), due to its longer 4 carbon linker. These two residues adopt a distinct conformation to accommodate the 14‐3‐3σ pocket, leading to the β‐homoSer(+2) side chain of **4** forming a direct hydrogen bond with K49 of 14‐3‐3, replacing a water‐mediated hydrogen bond as seen for **2** and **3** (Figure S5).

The cooperativity (*α*), a concentration‐independent parameter expressing the extent to which 14‐3‐3, the macrocycle and ChREBP enhance their mutual affinities to form the ternary complex, was evaluated using two‐dimensional (2D) FA titrations, by titrating 14‐3‐3 to **F1**–**F4** in the presence of varying ChREBP concentrations. The anisotropy values reached an elevated plateau at high ChREBP concentrations, due to the increased molecular volume of the ternary complex. At very high 14‐3‐3 concentrations, the anisotropy values decreased, or hooked, as the excess 14‐3‐3 favored the formation of binary complexes over ternary complexes. The combined binding curves were fitted to a thermodynamic cooperativity model, which also takes into account the sequential binding of two ChREBP sequences to the dimeric 14‐3‐3 platform (Figure 3a and Figures S6–S8; Table S4).^[^ ^25^ ^]^ The affinity of ChREBP for 14‐3‐3 was determined in previous work and fixed at *K*_D_^II^ = 1.5 µM in all fits.^[^ ^21^ ^]^ Macrocycle **F1** exhibited the highest cooperativity (*α* = 339 ± 35) for ternary complex formation, indicating that despite its relatively weak binding affinity for 14‐3‐3 (*K*_D_^I^ = 72 ± 1.4 µM), the formation of the ternary 14‐3‐3/ChREBP/**F1** complex is highly favored. Interestingly, as the binary affinity of the macrocycles for 14‐3‐3 increased, the cooperativity factor tended to decrease. For example, macrocycle **F4** resulted in an *α* factor comparable to **pepD** (*α* = 42 ± 1 and *α* = 38 ± 2, respectively), while macrocycle **F3**, which shows the highest binding affinity to 14‐3‐3, exhibited the lowest cooperativity (*α* = 12 ± 1). Notably, the range of cooperativity factors of the macrocycles mimics the scale of cooperativity achieved with seminatural products in PPI stabilization.^[^ ^26^ ^]^

**Figure 3:**
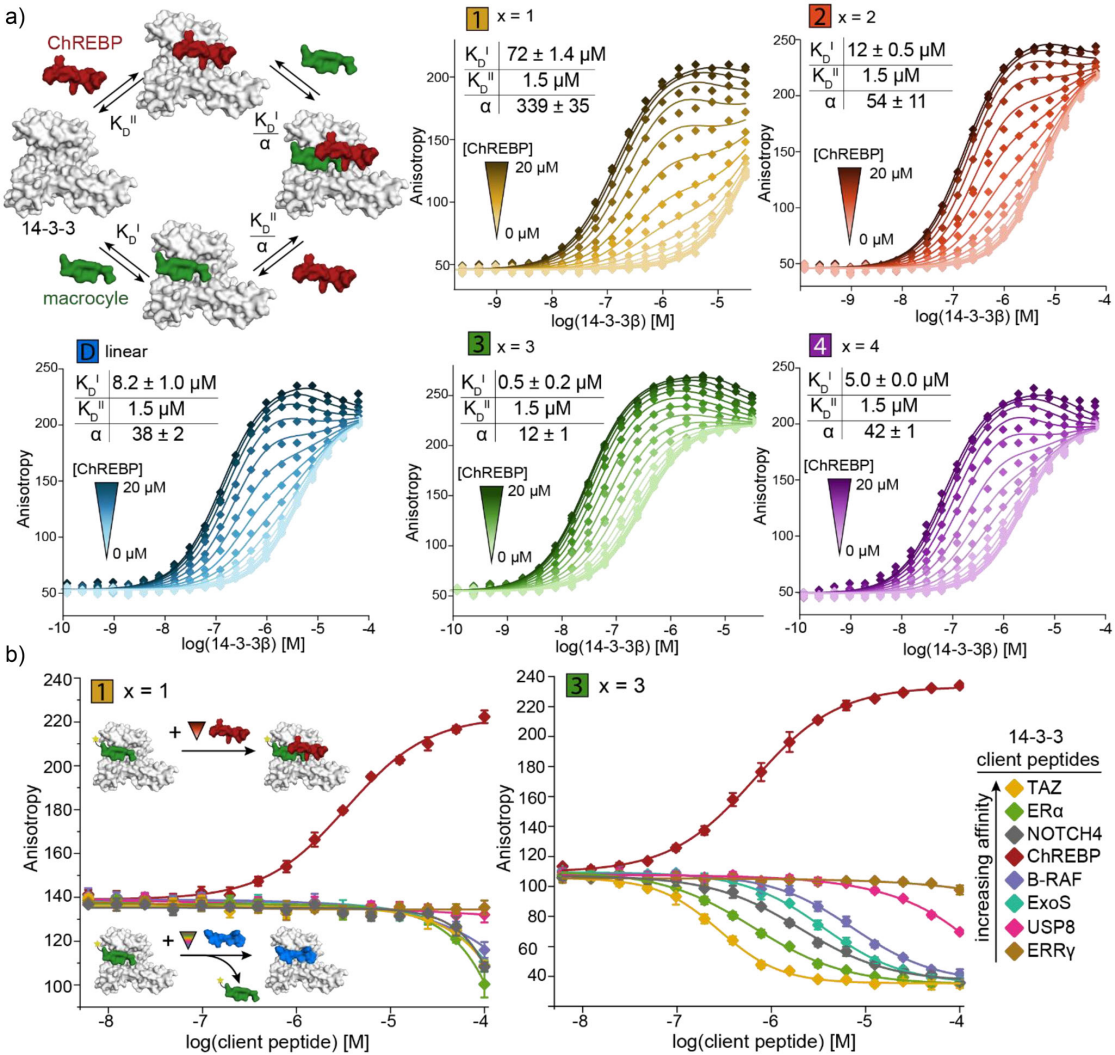
a) Simplified cooperativity scheme involving the sequential addition of macrocycle (affinity for 14‐3‐3 = *K*_D_^I^) and ChREBP peptide (affinity for 14‐3‐3 = *K*_D_^II^) to 14‐3‐3 or vice versa. For the full cooperativity scheme, see Figure S6, replicate data (*n* = 2) and fitting details, see Supporting Information. Plots represent 2D FA assays in which 14‐3‐3β is titrated to FITC‐labeled MG (**F1**, **F2**, **F3**, **F4**, **pepD**, 10 nM) in the presence of varying concentrations of ChREBP peptide. The thermodynamic model fitted the cooperativity (*α*) factor and *K*_D_^I^ with the 2D titration data and *K*_D_^II^ as input. b) Titration of acetylated 14‐3‐3 client peptides to 14‐3‐3β (concentration equal to EC_50_: 100 µM for **1**, 0.3 µM for **3**) and FITC‐labeled macrocycles **F1** and **F3** (10 nM). Stabilization of the 14‐3‐3β/macrocycle complex by ChREBP (red), while other peptides inhibit the complex (negative curves) (mean ± SD, *n* = 3).

An optimal 14‐3‐3 MG requires a balance between cooperativity and binary affinity for 14‐3‐3, without compromising the binding of other proteins to 14‐3‐3. To assess the selectivity of the macrocyclic MGs, a panel of eight known 14‐3‐3 client peptides with different binding modes and affinities was evaluated (Figure 3b). Gratifyingly, only the 14‐3‐3/ChREBP/macrocycle ternary complex formed selectively. The other client peptides, in contrast, inhibited the 14‐3‐3/macrocycle complex. Notably, the highly cooperative macrocycle **1** induces ternary complex formation at a ChREBP peptide concentration that is much lower than the concentrations required for inhibition of the 14‐3‐3/macrocycle complex by the other client peptides (Figures 3b and S9).

Previous attempts to co‐crystallize the ternary 14‐3‐3/ChREBP/**pepD** complex were unsuccessful.^[^ ^22^ ^]^ However, the more potent macrocycles **3** and **4** allowed crystallization of their ternary complexes. Clear electron density for the macrocycle and ChREBP peptide was observed in the 14‐3‐3 binding pocket (Figures 4a,b and S10a,b). Key interactions can be observed at the macrocycle/ChREBP interface, including a hydrogen bond between the macrocycle's phosphoserine backbone and N124 of ChREBP, as well as hydrogen bonds between the phosphate group and R128 of ChREBP (Figure 4c). These direct interactions likely account, in part, for the cooperativity of ternary complex formation. In addition to these macrocycle‐ChREBP interactions, both the macrocycles and ChREBP form contacts with the 14‐3‐3 protein, largely mirroring those detected in the corresponding binary complexes (Figures S5 and S10c–f). This is further illustrated by a crystallographic overlay of the macrocycles in their binary and ternary states, which reveals only subtle differences while maintaining critical contacts with 14‐3‐3 (Figure S10e–f).

**Figure 4:**
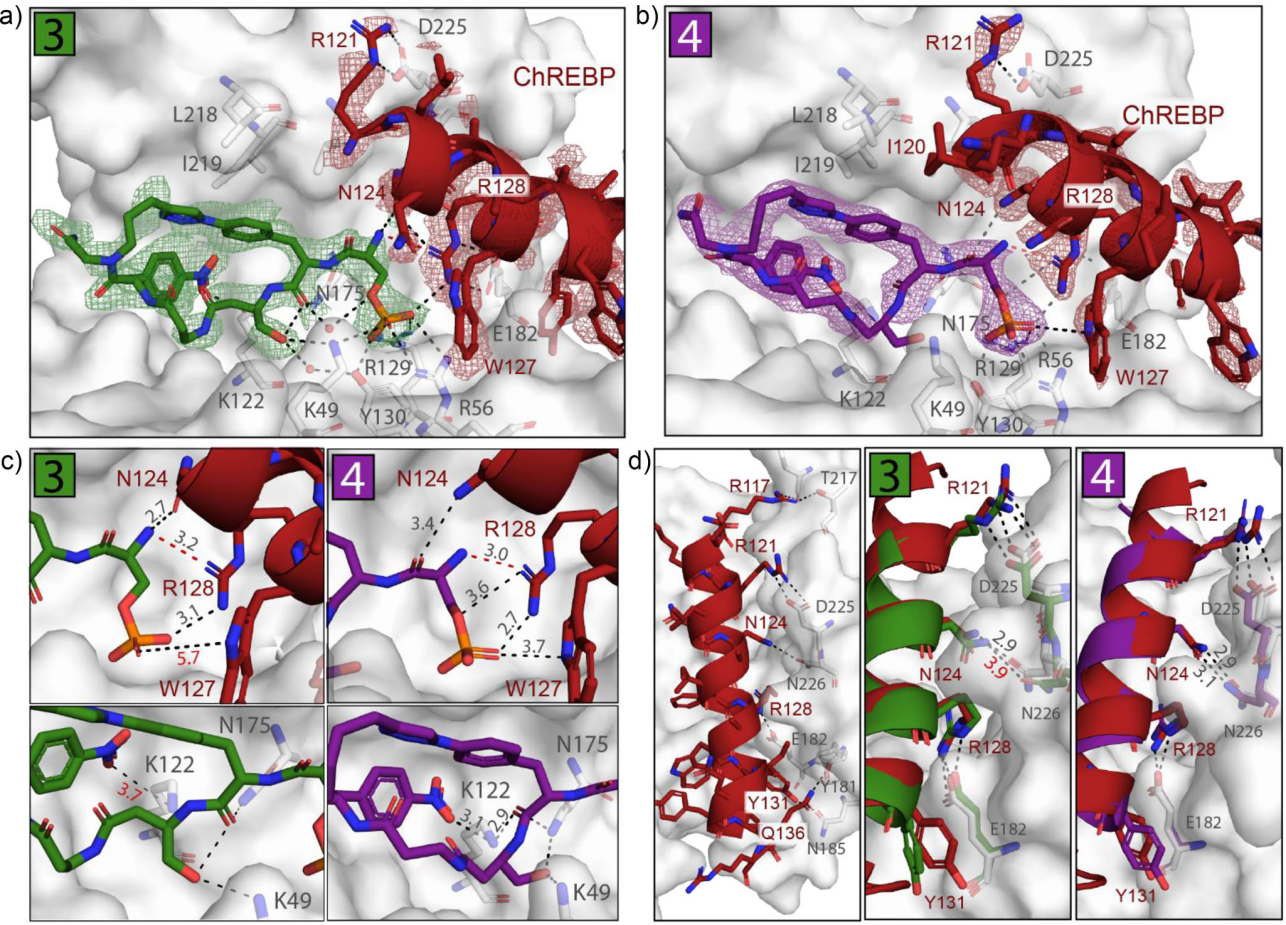
a,b) Ternary crystal structure of the 14‐3‐3σ protein (grey surface, relevant side chains are displayed as sticks) binding to macrocycle **3** (a) and **4** (b) (green and purple sticks) (polar contacts are shown as black dashed lines). Final 2*F*_o_−*F*_c_ electron density of macrocycles and ChREBP contoured at 1.0*σ*. c) Zoom‐in on key contacts between macrocycles and ChREBP (top) and 14‐3‐3 (bottom) (lengths of polar contacts in Å). d) 14‐3‐3/ChREBP interactions of the binary complex (left), in the presence of macrocycles **3** (middle) and **4** (right) (binary ChREBP = red, ternary ChREBP = green or purple upon **3** and **4** binding, respectively).

In‐depth comparison of the ternary crystal structures of macrocycles **3** and **4** also revealed notable differences. First, the phosphoserine bridging oxygen atom of **3** adopts a different orientation relative to its position in the binary complex, leading to the loss of a hydrogen bond with R128 of ChREBP that is maintained in the structure with **4** (Figure 4c). Second, although the phosphate of **4** establishes a hydrogen bond with W127 of ChREBP, this interaction is disrupted in the ternary complex with **3** due to the flipped side chain of W127. Furthermore, the hydrogen bond between Nph(+4) and K122 of 14‐3‐3 is weakened upon ternary complex formation with **3** (3.7 Å) compared to **4** (3.1 Å) (Figure 4c). Finally, binding of **3** also diminishes the interaction between N124 of ChREBP and N226 of 14‐3‐3, as N226 is moved away from ChREBP upon macrocycle **3** binding (Figures 4d and S11). Together, the loss of these interactions in the presence of macrocycle **3** likely forms the molecular basis for its reduced cooperativity compared to **4**.

This study presents the structure‐guided discovery of the first macrocyclic MGs stabilizing the 14‐3‐3/ChREBP PPI. In depth thermodynamic, kinetic, and structural studies revealed that the high binding affinity of the macrocycles results from reduced entropic penalties for complex formation in combination with design‐maintained molecular recognition with concomitant enthalpic contributions. The enhanced binding kinetically benefits from both a faster *k*_on_ and a reduced *k*_off_. Formation of the ternary 14‐3‐3/ChREBP/macrocycle complex is strongly cooperative, with an intriguing inverse affinity‐cooperativity correlation, for the different MGs. Structural analysis of the ternary complexes provides molecular explanations for these opposing effects, highlighting the conserved hydrogen bonds between ChREBP and the different macrocycles that likely drive cooperative assembly and the differences between **3** and **4** at the PPI interface, leading to the fine‐tuning of cooperativity. Selectivity assays testified to the preferential stabilization of the 14‐3‐3/ChREBP complex by the cooperative MGs over several other 14‐3‐3 client peptides. Evaluation of macrocycle cell permeability and cellular activity could potentially require matching with phosphate prodrug strategies.^[^ ^27^ ^]^ The findings reported provide a conceptual framework for the rational design of macrocyclic MGs, emphasizing the importance of thermodynamic and kinetic optimization as well as insights into the molecular basis and interplay of affinity and cooperativity in ternary complex formation, a potentially more broadly applicable MG phenomenon. These insights should pave the way for further refinement and other macrocycle‐based MGs that selectively modulate 14‐3‐3 PPIs and beyond.

## Conflict of Interests

C.O. and L.B. are co‐founders of Ambagon Therapeutics.

## Supporting information



Supporting Information

## Data Availability

The data that support the findings of this study are available in the Supporting Information of this article.
