# Supplementary material for: Macrocyclic Molecular Glues for the 14‐3‐3/ChREBP Interaction: Affinity and Cooperativity in an Inverse Relationship
Source: Angew Chem Int Ed Engl. 2025 Dec 18;65(6):e21678. doi: 10.1002/anie.202521678 (PMC12865133; doi:10.1002/anie.202521678)
Supplement: Supplementary file 1 — Supporting Information [file ANIE-65-e21678-s001.docx]

Supporting Information

**SUPPLEMENTARY TABLES**

**Table S1.** ITC parameters of experiments where 200 μM of macrocycle **3**, **4** and **pepD** is titrated to 20 μM of 14-3-3γ (mean ± SD, n=3).

| **Compound** | **n** | **ΔS (cal/mol)** | **ΔH (kcal/mol)** | **KD (µM)** | **ΔG (kcal/mol)** |
| --- | --- | --- | --- | --- | --- |
| 3 | 3 | 5.9 ± 0.89 | -7.5 ±0.70 | 0.167 ±0.10 | -9.24 ± 0.028 |
| 4 | 3 | 2.9 ± 0.78 | -7.4 ±0.44 | 1.05 ±0.18 | -8.15 ± 0.10 |
| pepD | 3 | -1.1 ± 1.0 | -7.9 ±0.39 | 2.78 ±0.70 | -7.58 ± 0.12 |

**Table S2**. SPR parameters of fitted data obtained by 2-fold dilution injections of macrocycle **3**, **4**, and **pepD** to Twinstrep-tagged 14-3-3γ captured on a StrepTactinXT chip (mean ± SD, n=2).

|  | | **Macrocycle 3** | | | **Macrocycle 4** | | |
| --- | --- | --- | --- | --- | --- | --- | --- |
|  |  | Replicate 1 | Replicate 2 | Average ± SD | Replicate 1 | Replicate 2 | Average ± SD |
| Kinetic fit | ka (1/Ms) | 6.29E+05 | 5.56E+05 | 5.9E+05 ± 5.4E+04 | 2.82E+05 | 2.90E+05 | 2.9E+05 ± 6.2E+03 |
|  | SE (ka) | 5.20E+03 | 1.50E+04 | - | 6.90E+03 | 3.30E+03 | - |
|  | kd (1/s) | 0.1384 | 0.1296 | 0.13 ± 0.01 | 0.3198 | 0.2988 | 0.31 ± 0.01 |
|  | SE (kd) | 0.0010 | 0.0034 | - | 0.0073 | 0.0032 | - |
|  | KD (M) | 2.20E-07 | 2.33E-07 | 2.3E-07 ± 0.1E-07 | 1.14E-06 | 1.03E-06 | 1.1E-06 ± 0.8E-07 |
|  | Rmax (RU) | 51.0 | 53.6 | - | 62.7 | 61.9 | - |
|  | tc | 2.92E+13 | 1.03E+11 | - | 4.07E+17 | 1.17E+18 | - |
|  | Chi² (RU²) | 0.832 | 0.957 | - | 4.67 | 1.12 | - |
|  | U-value | 3 | 3 | - | 9 | 4 | - |
| Affinity fit | KD (M) | 2.28E-07 | 2.53E-07 | 2.4E-07 ± 0.2E-07 | 1.18E-06 | 1.13E-06 | 1.2E-06 ± 0.4E-07 |
|  | Rmax (RU) | 50.7 | 52.6 | - | 61.8 | 61.4 | - |

**Table S2**. Continuation

|  | | **pepD** | | |
| --- | --- | --- | --- | --- |
|  |  | Replicate 1 | Replicate 2 | Average ± SD |
| Kinetic fit | ka (1/Ms) | 8.51E+04 | 8.23E+04 | 8.4E+04 ± 2.0E+03 |
|  | SE (ka) | 1.60E+03 | 2.9E+03 | - |
|  | kd (1/s) | 0.3375 | 0.3553 | 0.35 ± 0.01 |
|  | SE (kd) | 0.0059 | 0.0120 | - |
|  | KD (M) | 3.97E-06 | 4.32E-06 | 4.1E-06 ± 2.5E-07 |
|  | Rmax (RU) | 76.55 | 68.64 | - |
|  | tc | 1.30E+15 | 2.75E+15 | - |
|  | Chi² (RU²) | 4.67 | 12.6 | - |
|  | U-value | 7 | 12 | - |
| Affinity  fit | KD (M) | 3.86E-06 | 4.71E-06 | 4.3E-06 ± 0.6E-06 |
|  | Rmax (RU) | 75.4 | 66.4 | - |

**Table S3.** XRD data collection and refinement statistics for 14-3-3σ/macrocycle and 14-3-3/ChREBP/macrocycle structures.

| **PDB** | **9SA9** | **9SAA** | **9SAB** |
| --- | --- | --- | --- |
| Protein | 14-3-3σΔC | 14-3-3σΔC | 14-3-3σΔC |
| Macrocycle | **2** | **3** | **4** |
| Beam | ESRF ID23-2 | ESRF ID23-2 | ESRF ID30A-3 |
|  |  |  |  |
| *Data collection* |  |  |  |
| Wavelength (Å) | 0.873128 | 0.873128 | 0.967697 |
| Space group | P 21 21 21 | P 21 21 21 | P 21 21 21 |
| Cell dimensions  a, b, c (Å)  α, β, γ (°) | 63.31, 70.10, 128.17  90, 90, 90 | 63.44, 70.39, 128.31  90, 90, 90 | 63.38, 70.56, 128.31  90, 90, 90 |
| Resolution (Å) | 45.51 – 1.60  (1.62 – 1.60) | 64.15 – 1.50  (1.52 – 1.50) | 56.83 – 1.50  (1.52 – 1.50) |
| *I* / σ(*I)* | 18.5 (2.4) | 15.5 (1.4) | 13.3 (2.4) |
| Completeness (%) | 90.9 (100.0) | 100.0 (100.0) | 99.94 (100.0) |
| Redundancy | 13.2 (13) | 13.2 (13.0) | 14.2 (14.8) |
| CC_1/2_ | 0.999 (0.803) | 0.999 (0.626) | 0.997 (0.783) |
|  |  |  |  |
| *Refinement* |  |  |  |
| No. reflections | 69353 | 91424 | 92686 |
| R_work_/R_free_ | 0.181/0.206 | 0.172/0.207 | 0.184/0.207 |
| No. atoms  Protein  Ligand/ion  Water | 3669  125  404 | 3765  133  442 | 3656  127  532 |
| *B*-factors  Protein  Ligand/ion  Water | 27.26  54.20  34.68 | 26.41  32.39  35.94 | 21.10  28.72  31.65 |
| R.m.s. deviations  Bond lengths (Å)  Bond angles (°) | 0.019  1.32 | 0.016  1.30 | 0.017  1.26 |
| Ramachandran  favored (%)  outliers (%) | 99.12  0.00 | 98.70  0.00 | 99.11  0.00 |

**Table S3.** Continuation

| **PDB** | **9SAC** | **9SAE** |
| --- | --- | --- |
| Protein | 14-3-3σΔC | 14-3-3σΔC |
| Peptide | ChREBP | ChREBP |
| Macrocycle | **3** | **4** |
| Beam | ESRF ID23-2 | ESRF ID23-2 |
|  |  |  |
| *Data collection* |  |  |
| Wavelength (Å) | 0.873128 | 0.873128 |
| Space group | C 2 2 21 | C 2 2 21 |
| Cell dimensions  a, b, c (Å)  α, β, γ (°) | 82.48, 97.8, 81.45  90, 90, 90 | 82.87, 113.00, 63.47  90, 90, 90 |
| Resolution (Å) | 49.87 – 2.5  (2.75 – 2.5) | 56.5 – 2.3  (2.53 – 2.3) |
| *I* / σ(*I)* | 5.7 (2.5) | 5.5 (1.8) |
| Completeness (%) | 94.4 (99.7) | 92.72 (99.9) |
| Redundancy | 7.0 (7.0) | 7.1 (7.6) |
| CC_1/2_ | 0.687 (0.424) | 0.980 (0.502) |
|  |  |  |
| *Refinement* |  |  |
| No. reflections | 11003 | 12607 |
| R_work_/R_free_ | 0.288/0.356 | 0.239/0.288 |
| No. atoms  Protein  Ligand/ion  Water | 1918  60  135 | 1962  63  68 |
| *B*-factors  Protein  Ligand/ion  Water | 27.47  23.56  2.00 | 35.69  57.62  34.85 |
| R.m.s. deviations  Bond lengths (Å)  Bond angles (°) | 0.008  1.16 | 0.008  1.04 |
| Ramachandran  favored (%)  outliers (%) | 98.25  0.00 | 98.31  0.00 |

**Table S4.** Known and fitted parameters of the thermodynamic model to determine the cooperativity of the 14-3-3/ChREBP/macrocycle complex.

|  | Macrocycle **1** | | Macrocycle **2** | | Macrocycle **3** | | | Macrocycle **4** | | | Peptide **D** | | |
| --- | --- | --- | --- | --- | --- | --- | --- | --- | --- | --- | --- | --- | --- |
|  | Rep 1 | Rep 2 | Rep 1 | Rep 2 | | Rep 1 | Rep 2 | | Rep 1 | Rep 2 | | Rep 1 | Rep 2 |
| KDII (μM) (known) | 1.5 | 1.5 | 1.5 | 1.5 | | 1.5 | 1.5 | | 1.5 | 1.5 | | 1.5 | 1.5 |
| KDI (μM) | 71 | 73 | 12.6 | 11.9 | | 0.63 | 0.38 | | 5.0 | 5.0 | | 8.9 | 7.5 |
| α | 314 | 363 | 46 | 61 | | 12.3 | 11 | | 42.5 | 41.3 | | 39.8 | 36.8 |
| α1 | 82.8 | 85.4 | 18.8 | 8.3 | | 11.7 | 11 | | 11.8 | 13.5 | | 19.4 | 9.7 |
| Basis | 46 | 49 | 46 | 47 | | 55 | 51 | | 49 | 49 | | 53 | 54 |
| Gain  (1433) | 205 | 190 | 233 | 217 | | 222 | 213 | | 200 | 192 | | 211 | 200 |
| Gain2  (1433 1xS) | 208 | 198 | 245 | 230 | | 249 | 233 | | 208 | 199 | | 219 | 203 |
| Gain3  (1433 2xS) | 212 | 209 | 253 | 239 | | 272 | 271 | | 230 | 231 | | 240 | 230 |

**SUPPLEMENTARY FIGURES**


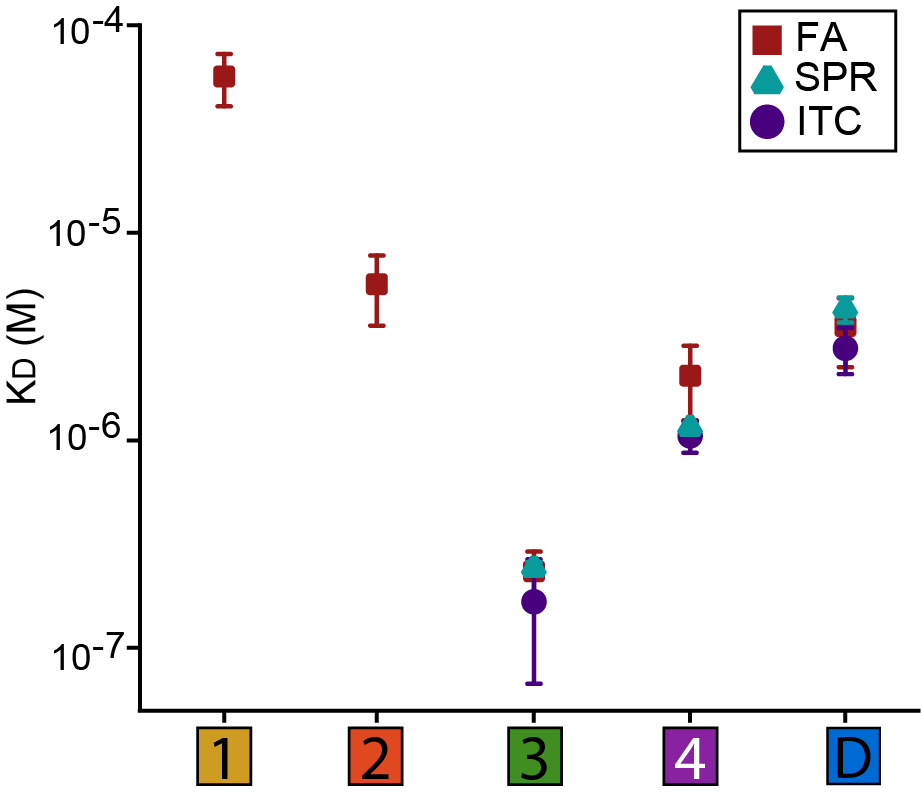


**Figure S1.** Overview of K_D_ values determined with FA, SPR and ITC for macrocycles **1**-**4** and **pepD**, revealing an optimal linker length of x=3 and consistent K_D_ values among methods.


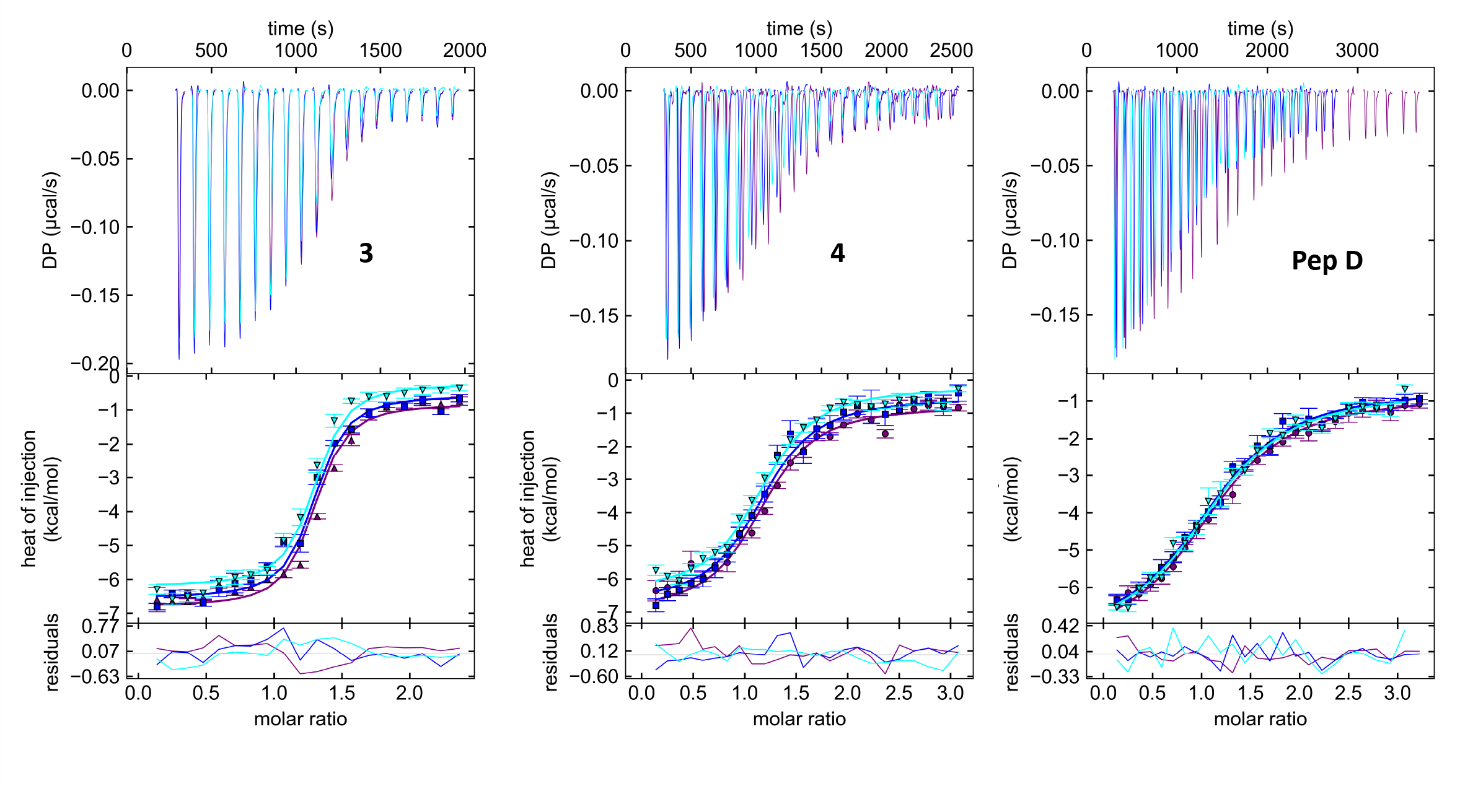


**Figure S2.** ITC data of 200 μM of macrocycle **3**, **4** and **pepD** titrations to 20 μM of 14-3-3γ (mean ± SD, n=3). Data is fitted with SEDPHAT software.


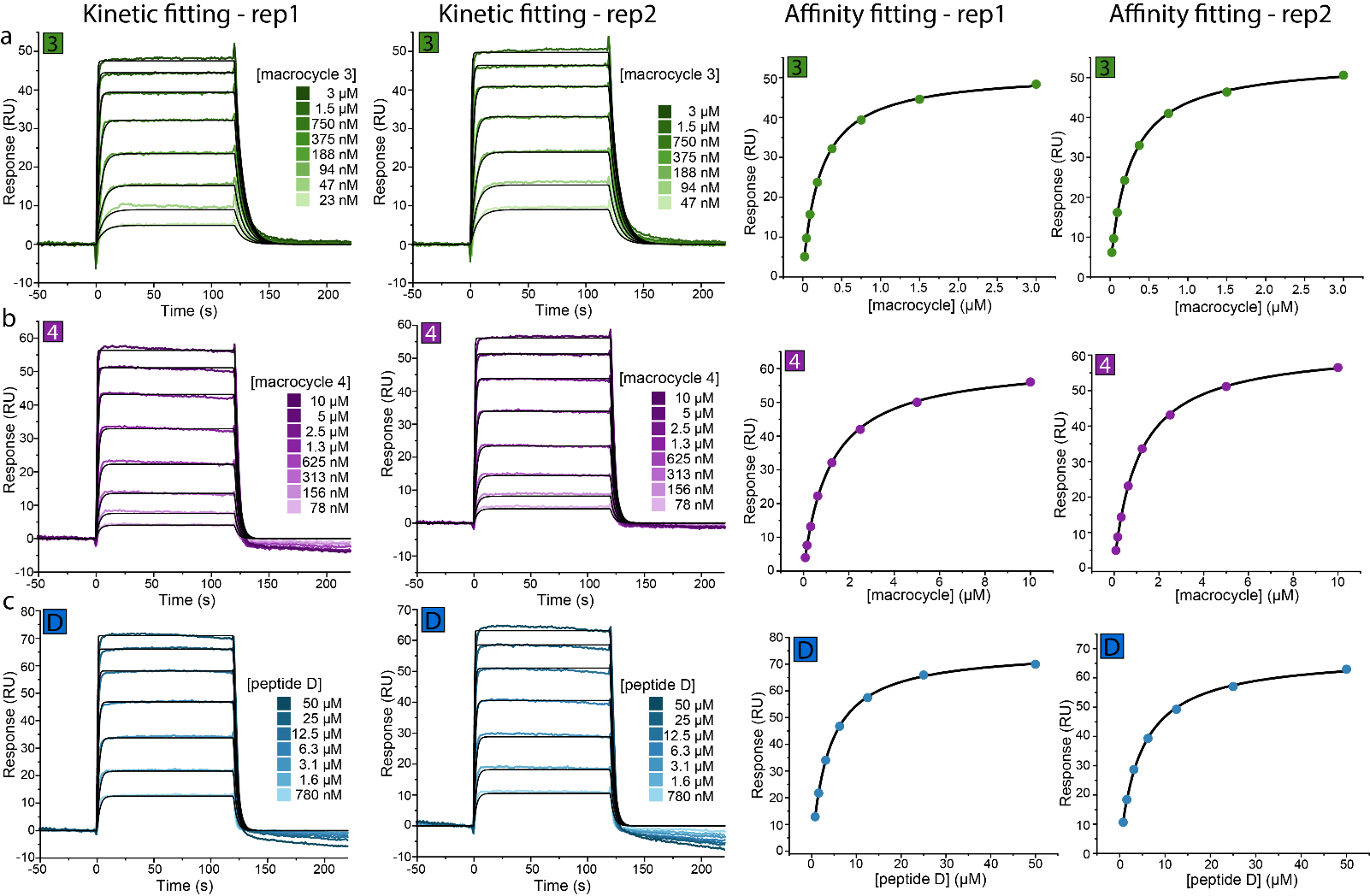


**Figure S3.** SPR data of 2-fold dilution injections of macrocycle **3** (a), **4** (b), and **pepD** (c) to Twinstrep-tagged 14-3-3γ captured on a StrepTactinXT chip (mean ± SD, n=2). Kinetic fits (left) and affinity fits (right) for two replicates.


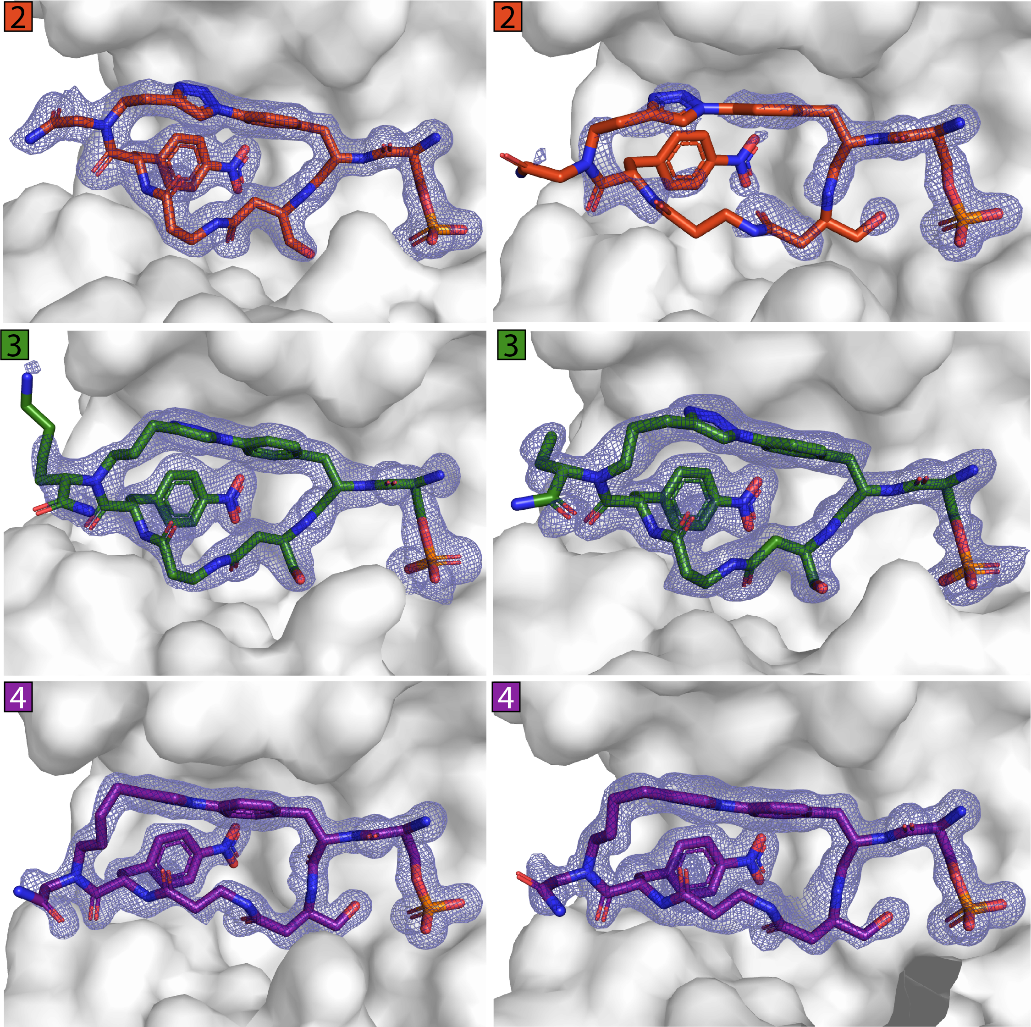


**Figure S4.** Crystal structures of the 14-3-3σ protein (grey, surface) binding to macrocycles **2** (orange), **3** (green), **4** (purple) of both 14-3-3 monomers found in the asymmetric unit (left and right). Final 2F_o_-F_c_ electron density contoured at 1.0σ.

**
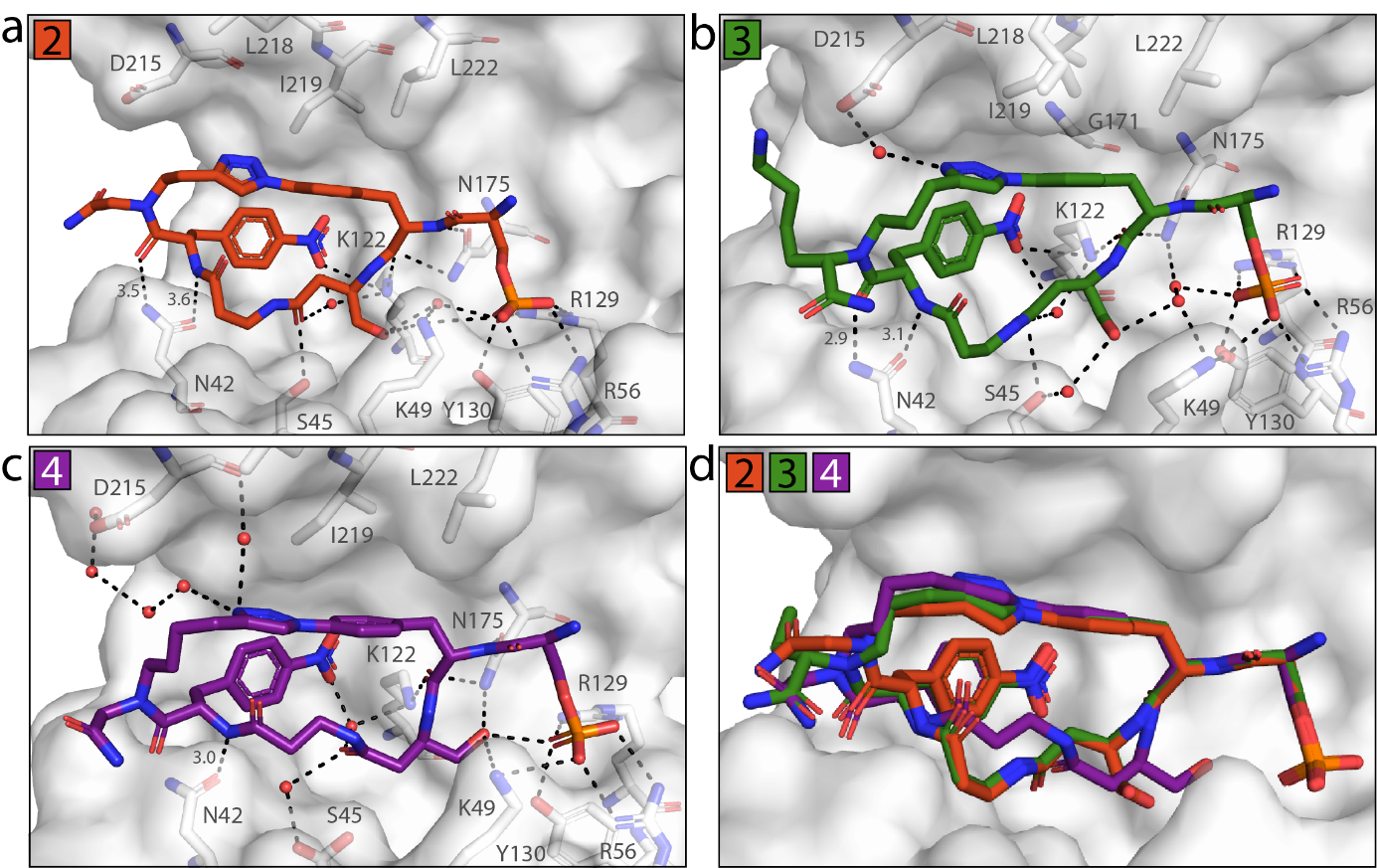
Figure S5.** Crystal structures of the 14-3-3σ protein (grey, surface) binding to macrocycles **2** (**a**) (orange), **3** (**b**) (green), **4** (**c**) (purple). Polar contacts are shown as black dashed lines. **d)** Crystallographic overlay of macrocycles **2**, **3**, and **4**.


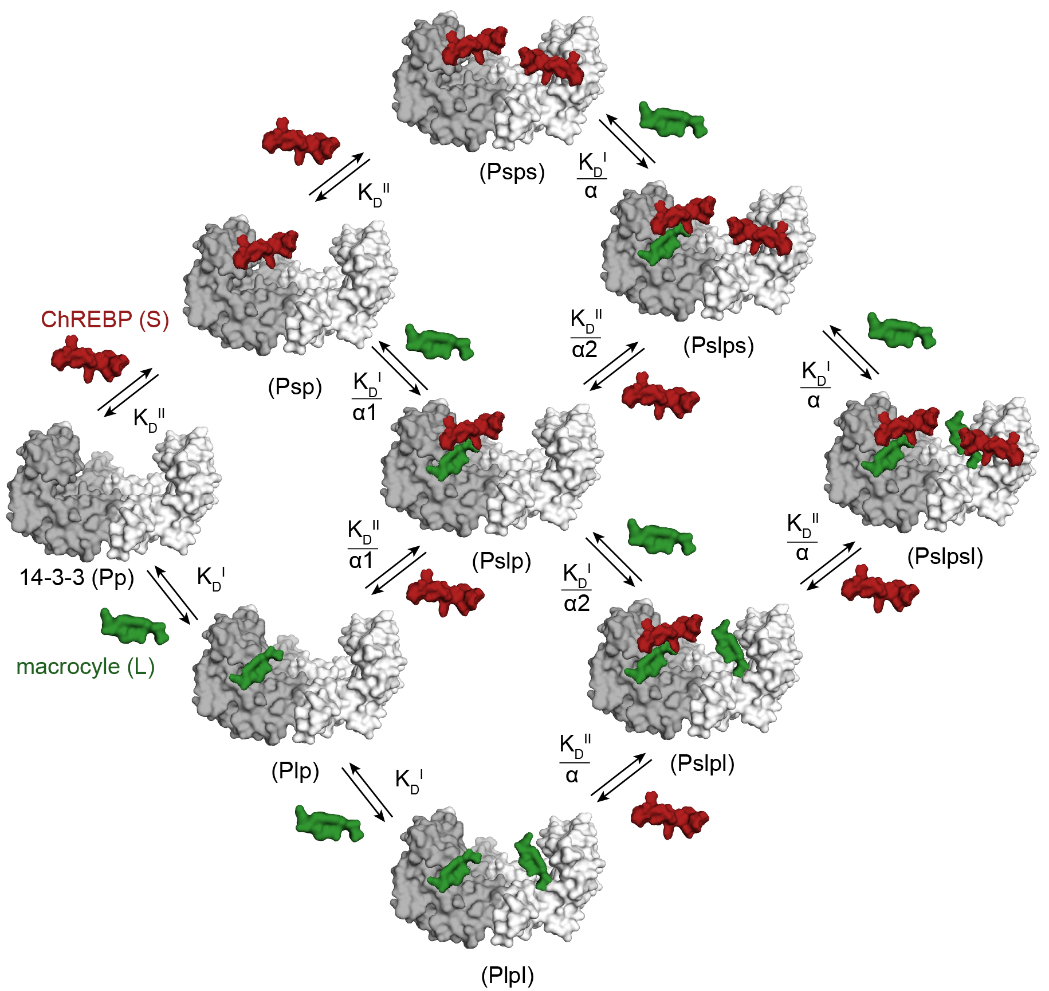


**Figure S6.** Complete representation of the species used in the thermodynamic model using the dimeric form of 14-3-3 (Pp) that can bind two macrocycle (L) species and two ChREBP (S) species.


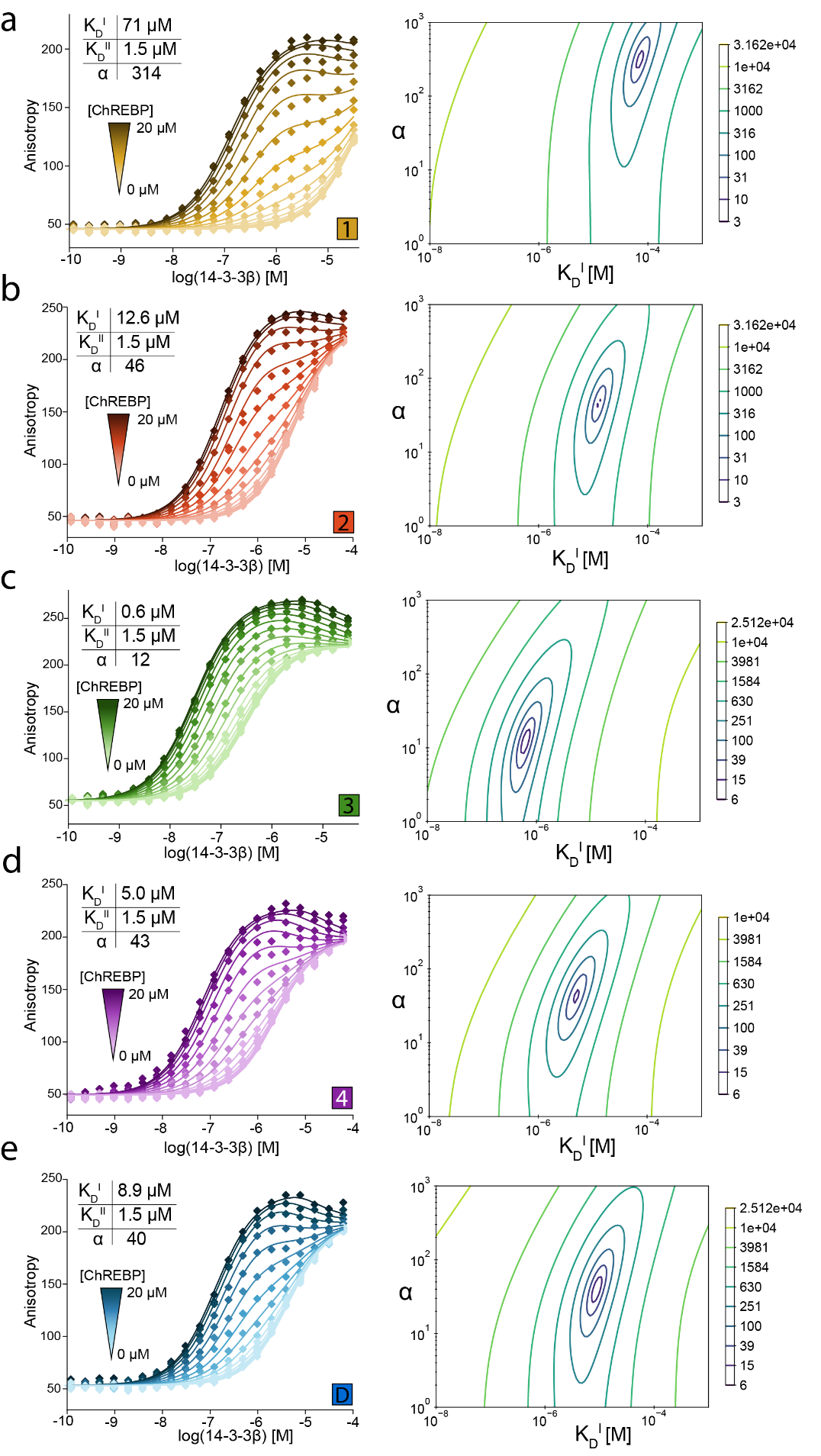


**Figure S7.** Replicate 1. Plots (left) represent 2D FA assays in which 14-3-3β is titrated to FITC-labeled macrocycle (**1**, **2**, **3**, **4**, **pepD**) in the presence of varying concentrations of ChREBP peptide, starting at 20 μM. Points represent measured anisotropy values, lines are fitted by the model. The α-factor and K_D_^I^ are fitted by the model and K_D_^II^ is used as known parameter. 2D mean squared error landscape plots (right) of model fits showing the minimum found for parameters α and K_D_^I^.


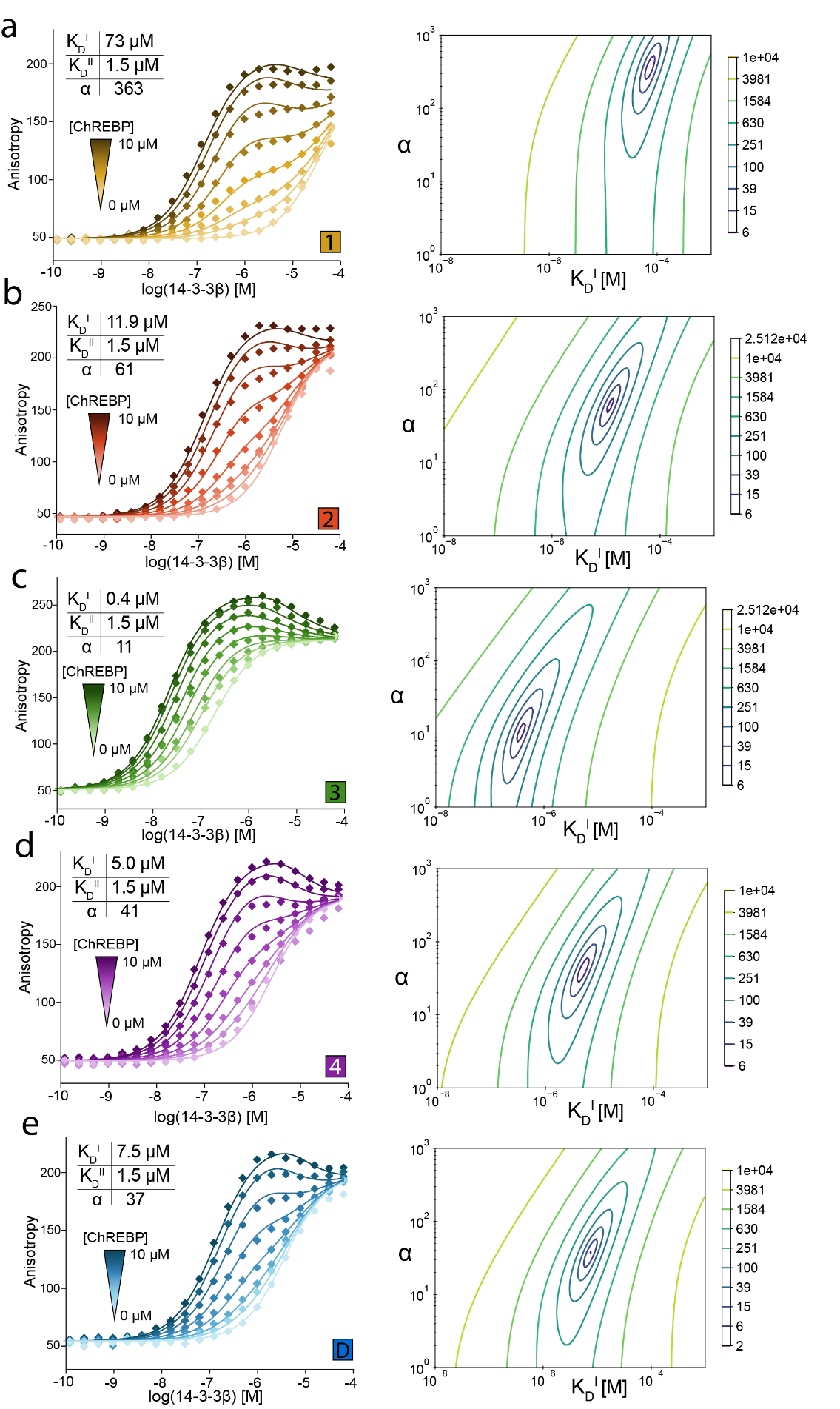


**Figure S8.** Replicate 2. Plots (left) represent 2D FA assays in which 14-3-3β is titrated to FITC-labeled macrocycle (**1**, **2**, **3**, **4**, **pepD**) in the presence of varying concentrations of ChREBP peptide, starting at 10 μM. Points represent measured anisotropy values, lines are fitted by the model. The α-factor and K_D_^I^ are fitted by the model and K_D_^II^ is used as known parameter. 2D mean squared error landscape plots (right) of model fits showing the minimum found for parameters α and K_D_^I^.


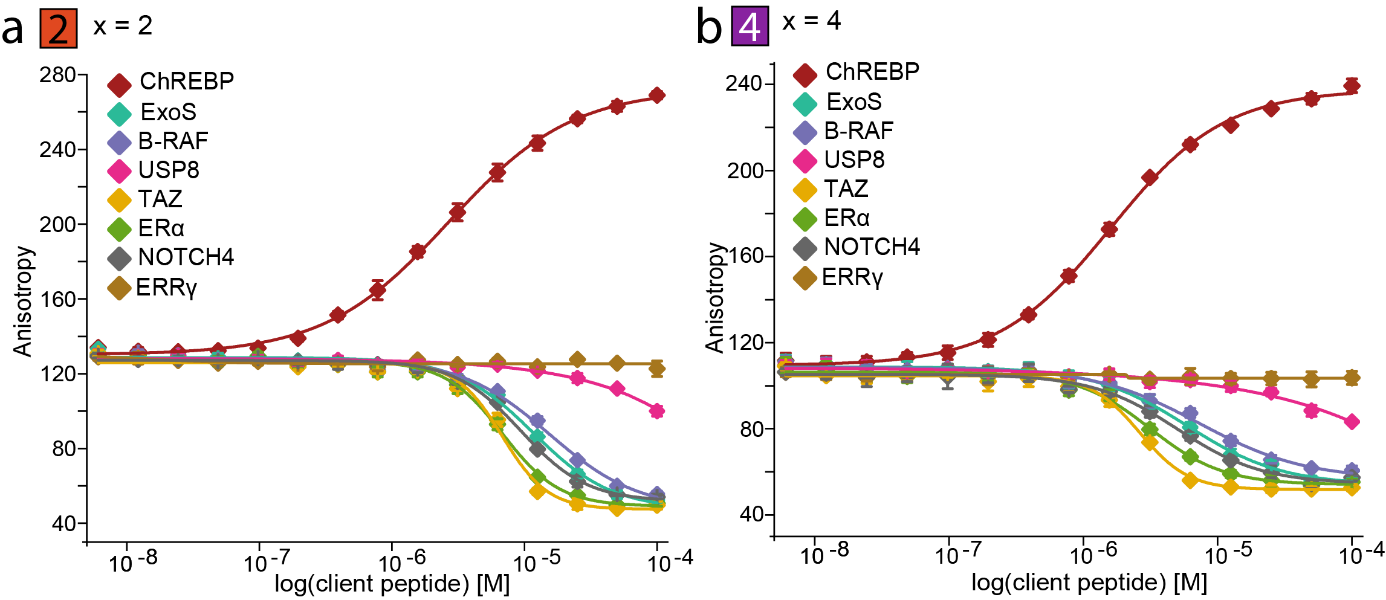


**Figure S9.** Titration of acetylated 14-3-3 client peptides to 14-3-3β (concentration equal to EC_50_: 7 μM for **2**, 2 μM for **4**) and FITC-labeled macrocycles **F2** and **F4** (10 nM). Stabilization of the 14-3-3β / macrocycle complex by ChREBP (red), while other peptides inhibit the complex (negative curves).

**
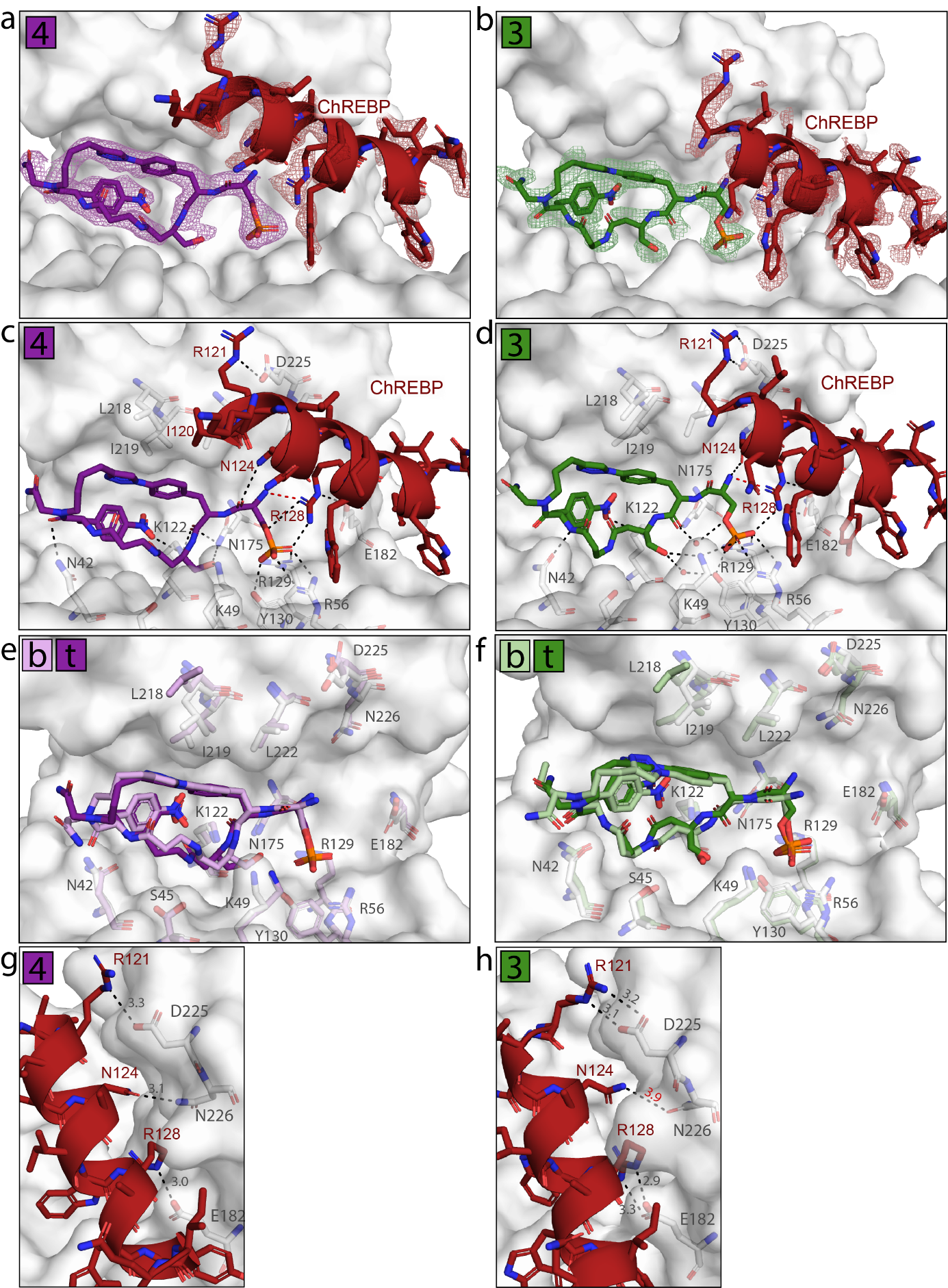
Figure S10. a**-**b**) Crystal structure of the 14-3-3σ protein (grey, surface) bound to macrocycle **4** (purple, sticks) / **3** (green, sticks) and the ChREBP peptide (red, cartoon, sticks). Final 2Fo-Fc electron density contoured at 1.0σ. **c**-**d**) Interactions of macrocycles 4 and 3 at the 14-3-3/ChREBP interface (relevant side chains are displayed as sticks). Polar contacts are shown as black dashed lines. Potential repulsive interactions as red dashed lines. **e**-**f**) Crystallographic overlay of the binary (b, light colors) and ternary (t, dark colors) structures for macrocycles **4** and **3**. The relevant 14-3-3 side chains are displayed as sticks (grey for ternary, light color for binary). **g**-**h**) Interactions of the ChREBP peptide with the 14-3-3 protein when macrocycles **4** and **3** are bound. Polar contacts are shown as black dashed lines and distances are in Å.

**
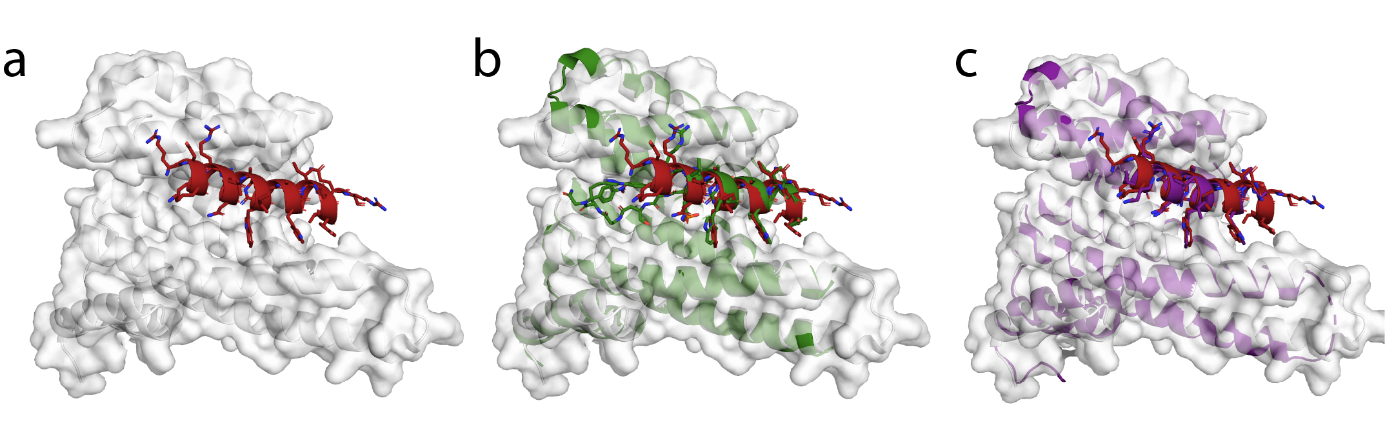
**

**Figure S11.** **a**) Crystal structure of the 14-3-3 (grey, surface, cartoon) / ChREBP (red, cartoon, sticks) binary complex with SO_4_ binding in the phospho accepting pocket (4GNT). **b**-**c**) Crystallographic overlays of the ternary crystal structures of 14-3-3/ChREBP/macrocycles **3** (green) and **4** (purple) with the binary 14-3-3 (grey) / ChREBP (red) structure.

**SUPPLEMENTARY METHODS**

Protein expression and purification

The full-length human 14-3-3β and γ proteins and the 14-3-3σ ΔC protein with a N-terminal His6 tag were expressed in BL21(DE3) competent Escherichia coli cells from a pPROEX HTb expression vector. Cells grew at 37 ̊C until absorption at 600 nm reached 0.8. Protein expression was induced with the addition of isopropyl β-D-1-thiogalactopyranoside (IPTG) to a final concentration of 1 mM, cells were incubated overnight at 18 ̊C before being collected and resuspended in 10 mL lysis buffer (50 mM HEPES pH=8.0, 300 mM NaCl, 12.5 mM imidazole, 5 mM MgCl2, 2 mM BME) per gram pellet. The cells were homogenized at a pressure of 40bar using Emulsiflex-C3 homogenizer. The homogenized mixture was centrifuged at 20.000xg for 20 min at 4 ̊C and the supernatant was loaded onto a nickel-nitrilotriacetic acid affinity column, pre-equilibrated with wash buffer (50 mM HEPES pH=8.0, 300 mM NaCl, 25 mM imidazole, 2 mM BME). After washing the column with wash buffer, the bound protein was eluted with 250 mM imidazole. The resulting protein complex was placed in a dialysis bag (10,000 Da pores) and placed in dialysis buffer (25 mM HEPES pH=8.0, 200 mM NaCl, 10 mM MgCl2, 2 mM BME, 5% glycerol). Protein concentration was measured using a Nanodrop-1000, analyzed for purity by SDS-PAGE and Q-Tof LC/MS, and the protein was aliquoted, flash frozen and stored at -80 ̊C. The ΔC variant was truncated at the C-terminus after T231 to enhance crystallization. After the first Ni-affinity chromatography column, the construct was treated with TEV protease to cleave off the His6 tag during dialysis (25 mM HEPES, pH 7.5, 200 mM NaCl, 5% glycerol, 10 mM MgCl_2_, 250 µM TCEP) overnight at 4 °C. The flow-through of a second Ni-affinity column was subjected to a final purification step by size exclusion chromatography (Superdex 75 pg 16/60 size exclusion column (GE Life Science) (SEC buffer: 25 mM HEPES pH 7.5, 100 mM NaCl, 10 mM MgCl_2_, 250 µM TCEP). The protein was concentrated to ~60 mg/mL, analyzed for purity by SDS-PAGE and Q-Tof LC/MS and aliquots flash-frozen for storage at -80 °C.

Peptide sequences

The acetylated ChREBP peptide and 14-3-3 client peptide panel used for selectivity studies were purchased from GenScript Biotech Corp. with the following sequences:

ChREBP: Ac-RDKIRLNNAIWRAWYIQYVKRRKSPV-CONH_2_, ExoS: Ac-QGLLDALDLAS-CONH_2_ B-RAF: Ac-RDRSS(pS)APNVH-CONH_2_, USP8: Ac-KLKRSY(pS)SPDITQ-CONH_2_, TAZ: Ac-HVRSH(pS)SPASLQLG, ERα: Ac-EGFPA(pT)V-COOH, NOTCH4: Ac-RPRTQ(pS)APHRRRPP-CONH_2_ , ERRγ: Ac-KRRRK(pS)CQA-CONH_2_.

Fluorescence Anisotropy assay

To measure binding affinity, 14-3-3γ was titrated with a starting concentration of 250 µM in a 2-fold dilution series against 5 nM of fluorescein-labeled macrocycle **F1**-**F4** in FP buffer (10 mM HEPES, pH 7.4, 150 mM NaCl, 0.1% (v/v) Tween20, 0.1% (w/v) BSA). For the 2D titrations, 14-3-3β was titrated in a 2-fold dilution series (starting at 250 µM) to a mix of fluorescein-labeled macrocycle (5 nM) against varying fixed concentrations of ChREBP (2-fold dilution starting from 20 µM (replicate 1) or 10 µM (replicate 2)). For the selectivity assay, acetylated 14-3-3 client peptides were titrated with a starting concentration of 100 μM in a 2-fold dilution series, including ChREBP, ExoS, B-RAF, USP8, TAZ, ERα, NOTCH4, ERRγ, to 10 nM of fluorescein-labeled macrocycle (**F1**-**F4**) bound to 14-3-3β (concentration around its EC_50_ value). These 14-3-3β concentrations were 100 μM for **1**, 7 μM for **2**, 0.3 μM for **3**, 2 μM for **4**.

Dilution series were made in a polystyrene (non-binding) low-volume Corning Black Round Bottom 384-well plates (Corning 4514 or 4511). Measurements were performed directly after plate preparation, using a Tecan Infinite F500 plate reader at room temperature (lex: 485 ± 20 nm; lem: 535 ± 25 nm; mirror: Dichroic 510; flashes: 20; integration time: 50 ms; settle time: 0 ms; gain: optimal; and Z-position: calculated from well). Wells containing 5 nm FITC-macrocycle were used to set as G-factor. All data were analyzed using Origin2023b and fitted using a four-parameter logistic model. Each measurement was performed in three independent experiments. Data and errors represent mean ± SD, for binding affinity and selectivity assays n=3, for 2D titrations n=2.

Cooperativity model

The cooperativity parameters for macrocycles **1**-**4** and **pepD** for 14-3-3/ChREBP stabilization were determined by using the thermodynamic equilibrium system as described previously, which is elaborated using the dimeric 14-3-3 protein (Fig. S6).^22^ The data from 2D-titrations was provided to the model including Kd2: 1.5 µM, P_tot = 5 nM, and the variable concentrations of 14-3-3 and ChREBP at each data point. Fit parameters were given the following initial guess values: Kd1: 12e-6, alpha: 60, alpha1: 15, basis: 47, gain: 220, gain2: 225, gain3: 240.

Species used in the model:

Pp = 14-3-3 dimer

S = ChREBP

L = macrocycle **1**-**4** / **pepD**


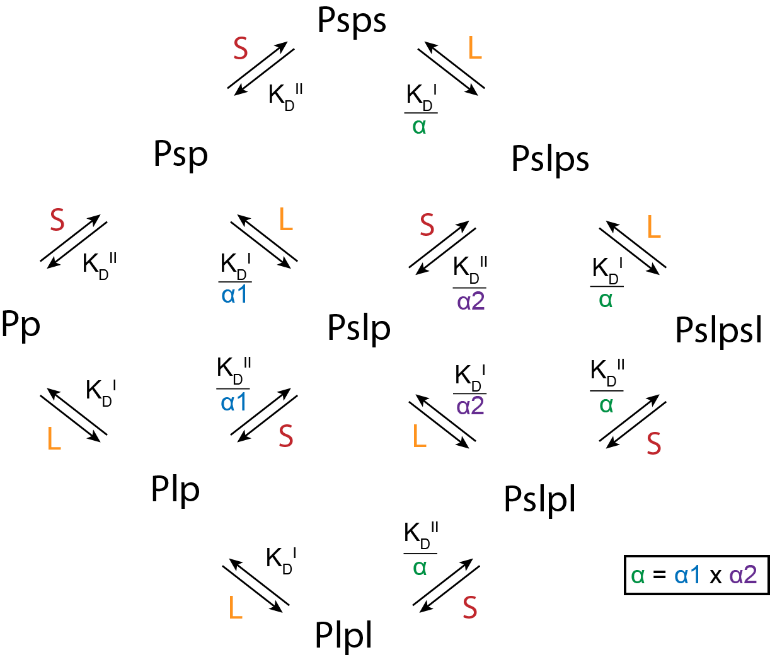
Equations:

Pp + S = Psp; Kd2

Pp + S = Pps; Kd2

Psp + S = Psps; Kd2

Pp + L = Plp; Kd1

Pp + L = Ppl; Kd1

Plp + L = Plpl; Kd1

Psp + L = Pslp; Kd1/alpha1

Pps + L = Ppsl; Kd1/alpha1

Pps + L = Plps; Kd1/(alpha/alpha1)

Psp + L = Pspl; Kd1/(alpha/alpha1)

Pslp + S = Pslps; Kd2/(alpha/alpha1)

Pslp + L = Pslpl; Kd1/(alpha/alpha1)

Ppsl + S = Pspsl; Kd2/(alpha/alpha1)

Ppsl + L = Plpsl; Kd1/(alpha/alpha1)

Psps + L = Pspsl; Kd1/(alpha)

Psps + L = Pslps; Kd1/(alpha)

Pslps + L = Pslpsl; Kd1/(alpha)

custom input: (L*basis)/L_tot + (Plp+Ppl+2*Plpl)*gain/L_tot + (Plps+Pspl+Pslp+Ppsl+2*Plpsl+2*Pslpl)*gain2/L_tot + (Pslps+Pspsl+2*Pslpsl)*gain3/L_tot

Isothermal Titration Calorimetry

Final dialysis fluid from protein expression was frozen as 2 mL aliquots to serve as ITC buffer (25 mM HEPES pH 8.0, 100 mM NaCl, 10 mM MgCl2, 500 µM TCEP). 14-3-3γ and peptide were dissolved and diluted in this buffer to 20 μM and 200 μM, respectively. Samples were degassed for 10 min prior to measurement at 450 mmHg. The reference cell was filled with 300 µL degassed MilliQ and the sample cell was filled with 300 µL of peptide or protein mixture. Syringe was loaded with at least 200 µL protein or peptide sample. Measurements were performed on an Affinity ITC LV (TA instruments), with injection size set to 2 µL, stirring speed of 125 rpm and temperature at 25 ̊C. The baseline was automatically adjusted with NITPIC, analysis of data was done in SEDPHAT, and images were produced with GUSSI software. Data and errors represent mean ± SD, n=3 replicates.

Surface Plasmon Resonance

The SPR experiments were performed at 25 °C using a Biacore X100 (Cytiva) and a 200 nm Strep-Tactin XT derivatized linear polycarboxylate hydrogel chip, medium charge density (XanTec Bioanalytics). All proteins and peptides were dissolved in fresh running buffer prepared with ultrapure water and filtered through a 0.2 μm filter (10 mM HEPES pH 7.4, 200 mM NaCl, 50 μM EDTA, 0.005% P20). First the surface was conditioned with a 1 min injection of 3 M Guanidine HCl. The Biacore X100 was primed with running buffer. Then, the recombinant 14-3-3γ-Twinstrep protein (250 nM) was captured on flow cell 2 of the sensor chip at a flow rate of 10 μL/min for 2 minutes, which resulted in a capture level of 1000 RU. Flow cell 1 was left blank as a reference surface. Multi-cycle kinetic measurements were conducted at a flow rate of 30 μL/min. A 2-fold dilution series of analyte (Ac-macrocycles **3**, **4** and **pepD**) in running buffer was injected over the sensor chip for 2 min, followed by dissociation for 2 min until full dissociation of the peptide. The highest concentration of analytes used depends on their affinity, see Fig. S3. Between cycles of one multi-cycle measurement, no regeneration step was performed due to the complete dissociation of the analyte. After a measurement, the chip was regenerated by 2 times 30 sec injections at flow rate 10 μL/min of 3 M Guanidine HCl. The data collection rate of the instrument is 1 datapoint/sec. The data was corrected by double subtracting to the reference surface (flow cell 1) and buffer injection (blank) and analyzed using 1:1 interaction fitting model with the BIA evaluation software (2020). All parameters were fitted using global fitting, including the Rmax, because a single immobilization was used for all analyte concentrations. The RI was set constant at 0 RU. Next to kinetic fitting, the affinity fit was made using a 1:1 binding model by plotting the response (RU) at steady state over the analyte concentration (Fig. S2).

X-ray crystallography^5^

14-3-3σ∆C/2-4: Complexes were made with 10-12 mg/ml 14-3-3σ∆C (truncated after T231 to reduce flexibility) and macrocyclic peptide in a ratio of 1:2 in complexation buffer (25 mM HEPES, pH 7.4, 100 mM MgCl_2_, and 2 mM BME). Crystals grew with the sitting drop method with a precipitation buffer containing 26-28% (v/v) PEG400, 5% glycerol, 0.2 M CaCl_2_ , 0.1 M HEPES pH 7.4. Crystals grew within 3 days. They were fished and flash-frozen before being measured on ESRF beamline, ID23-2 (**2**, **3**), ID30A-3 (**4**). 14-3-3σ∆C/**3**-**4**/ChREBP: Complexes were made with 10-12 mg/ml 14-3-3σ∆C, ChREBP peptide and macrocyclic peptide in a ratio of 1:2:2 in complexation buffer (25 mM HEPES, pH 7.4, 100 mM MgCl2 , and 2 mM BME). Crystals grew with the sitting drop method with a precipitation buffer containing 26-28% (v/v) PEG400, 5% glycerol, 0.2 M CaCl_2_, 0.1 M HEPES pH 7.4. Crystals grew within 14 days. They were fished and flash-frozen before being measured on ESRF beamline ID23-2. All Data was processed using the CCP4i2 suite (version 8.0.018) X2DIALs was used to index and integrate the data after which scaling was done using AIMLESS.^1^ The data was phased with MolRep, using protein data bank (PDB) entry 7ZMU as a template.^2^ A three-dimensional structure of macrocyclic peptides was generated using AceDRG17, which was thereafter built in based on visual inspection with the help of the electron density map. Sequential model building and refinement were performed with COOT and REFMAC respectively.^3,4^

**Organic Synthesis and Characterization**

General Information (chemicals, materials, instrumentation)

All reactions were prepared using analytical grade (AR) grade solvents. All reagents were purchased from TCI, or Sigma-Aldrich and were used without further purification. Preparative HPLC was performed using a Gemini S4 110A 150 x 21.20 mm column using miliQ water with 0.1% formic acid (FA) and acetonitrile (ACN) with 0.1% FA. Analytical (LR) HPLC-MS analysis was performed on a system comprising a C4 Jupiter SuC4300A 150 x 2.0 mm column using miliQ water with 0.1% FA and acetonitrile with 0.1% FA, using a gradient of 5% to 100% ACN over 10 minutes, connected to a Thermo Fisher LTQ XL Linear Ion Trap Mass Spectrometer. The purity of the samples was assessed using PDA (280 nm) and MS (positive mode, m/z 100 – 1000). Unless otherwise stated all final compounds were ≥95% pure as judged by HPLC. High resolution mass spectra (HRMS) were recorded using a Waters ACQUITY UPLC I-Class LC system coupled to a Xevo G2 Quadrupole Time of Flight (Q-TOF) mass spectrometer equipped with a Phenomex kinetex® 2.6 μm EVO C18 100 x 2.1 mm column.

General Procedures

GP1: Amino acid coupling

Peptides were synthesized in 12 mL syringes. Fmoc-protected Rink resin (loading 0.51 mmol/g) was swelled in DMF (3.0 mL) for 30 min. The resin was drained, washed twice with DMF and treated with 20% piperidine in DMF for 2x5min to remove the Fmoc protecting group. The resin was drained and washed six times before the addition of Fmoc-protected amino acid (4.0 eq), HCTU (4.0 eq) and DIPEA (8.0 eq) dissolved in DMF (3.0 mL). The mixture was shaken for 1h before draining and washing (6x) the resin. Subsequently, Fmoc-group removal was conducted by treatment with 20% piperidine in DMF for 2x5 min at (room temperature) rt before coupling of the next amino acid.

GP2: Ns protection and Mitsunobu reaction

Attachment of the 2-nitrobenzenesulfonamide (Ns) was performed by the addition of 2-NsCl (3 eq) and 2,4,6-collidine (3 eq) in DCM (3 mL), the mixture was shaken for 16h at rt before draining and washing (6x) the resin. Mitsunobu reaction was carried out with the desired alcohol (6 eq), PPh_3_ (6 eq) and DIAD (6 eq) in THF for 16h at rt. After the reaction, the resin was washed and drained (6x). The Ns-group was then cleaved with 2-mercaptoethanol and DBU (10 v/v%) for 1h before draining and washing (6x) the resin.

GP4: Ring-closing CuAAC

Cyclization was performed on-resin by addition of Cu(MeCN)_4_PF_6_ (0.5 eq) TBTA (0.5 eq) and 2,4,6-collidine (2 eq) in purged DMF (3mL) for 16h at rt. After monitoring via LC-MS indicated completion of the reaction, the resin was washed and drained (6x)

GP5: Peptide cleavage and purification

The peptides were cleaved from the resin with a cleavage cocktail of TFA/Tis/H_2_O 95:2.5:2.5 (3 mL) for 2 hours. After cleavage, peptides were precipitated in a 1:1 mixture of cold pentane and ether and before spinning down at 4000 rpm for 10 minutes. The pellet was collected. The peptides were purified with preparative LC-MS with a 12%-17% ACNin H_2_O gradient.

GP6: Alloc-deprotection and FITC-coupling

The Alloc-protecting group was removed using Tetrakis(triphenylphosphine)palladium(0) (5 eq) and phenylsilane (5 eq) in DCM for 2x45 min. After washing and draining the resin (6x), a mixture of fluorescein isothiocyanate (FITC) (5 eq) was and DIPEA (10 eq) was added and the resin was shaken for 16h after which the resin was washed and drained (6x).

Detailed Synthetic Procedures and Characterization

F1

Peptide **F1** was synthesized using GP1 to couple Fmoc-Lys(Alloc)-OH, Fmoc-β-Ala-OH and Fmoc-Lys(Boc)-OH to the Rink amide resin (200 mg, 0.1 mmol) , after which GP2 was carried out with propargyl alcohol (**5**) (6 eq, 0.6 mmol, 33.6 mg). The linear peptide was further extended following GP1. Subsequently, the ring-closing reaction was performed using GP4 before removal of the Alloc protecting group and coupling of fluorescein through GP6, afterwards the peptide was deprotected and cleaved from the resin and purified according to GP5. After the purified fractions were collected and lyophilized, **F1** (0.3 mg, 0.2%) was obtained as a white amorphous powder.

LCMS (ESI): *m/z* 1492 ([M + H]^+^) t_R_ 3.01 min. HRMS (ESI): m/z = 746.2635 calculated for [C67H79N16O20PS + 2H]^2+^; found: 746.2631.


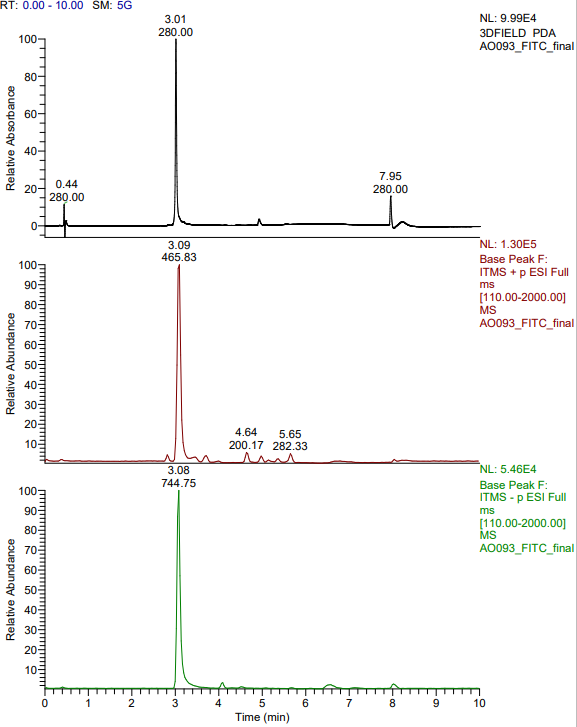


F2

Peptide **F2** was synthesized using GP1 to couple Fmoc-Lys(Alloc)-OH, Fmoc-β-Ala-OH and Fmoc-Lys(Boc)-OH to the Rink amide resin (200 mg, 0.1 mmol) , after which GP2 was carried out with 3-butyn-1-ol (**6**) (6 eq, 0.6 mmol, 35.7 mg). The linear peptide was further extended following GP1. Subsequently, the ring-closing reaction was performed using GP4 before removal of the Alloc protecting group and coupling of fluorescein through GP6, afterwards the peptide was deprotected and cleaved from the resin and purified according to GP5. After the purified fractions were collected and lyophilized, **F2** (3.4 mg, 2.3%) was obtained as a white amorphous powder.

LCMS (ESI): *m/z* 1505 ([M + H]^+^) t_R_ 2.97 min. HRMS (ESI): m/z = 753.2714 calculated for [C68H81N16O20PS + 2H]^2+^; found: 753.2712


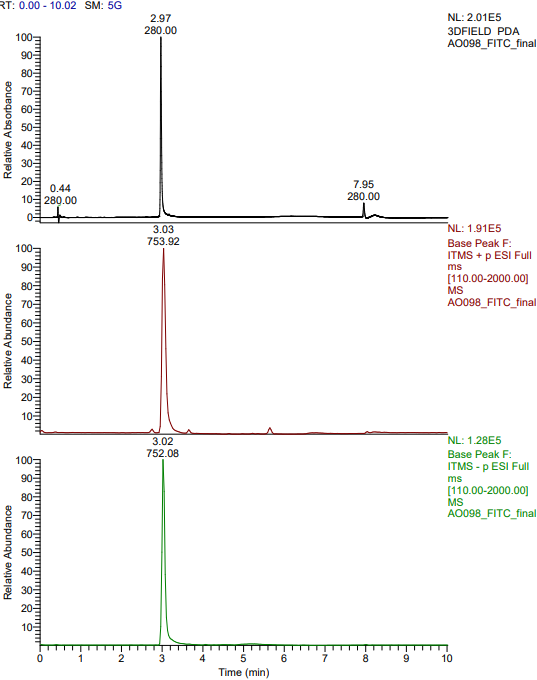


F3

Peptide **F3** was synthesized using GP1 to couple Fmoc-Lys(Alloc)-OH, Fmoc-β-Ala-OH and Fmoc-Lys(Boc)-OH to the Rink amide resin (200 mg, 0.1 mmol), after which GP2 was carried out with 4-pentyn-1-ol (**7**) (6 eq, 0.6 mmol, 42.9 mg). The linear peptide was further extended following GP1. Subsequently, the ring-closing reaction was performed using GP4 and the peptide was deprotected and cleaved from the resin and purified according to GP5. After the purified fractions were collected and lyophilized **F3** (5.1 mg, 3.4%) was obtained as a white amorphous powder. LCMS (ESI): *m/z* 1519 ([M + H]^+^) t_R_ 2.98 min. HRMS (ESI): m/z = 760.2792 calculated for [C69H83N16O20PS + 2H]^2+^; found: 760.3215.


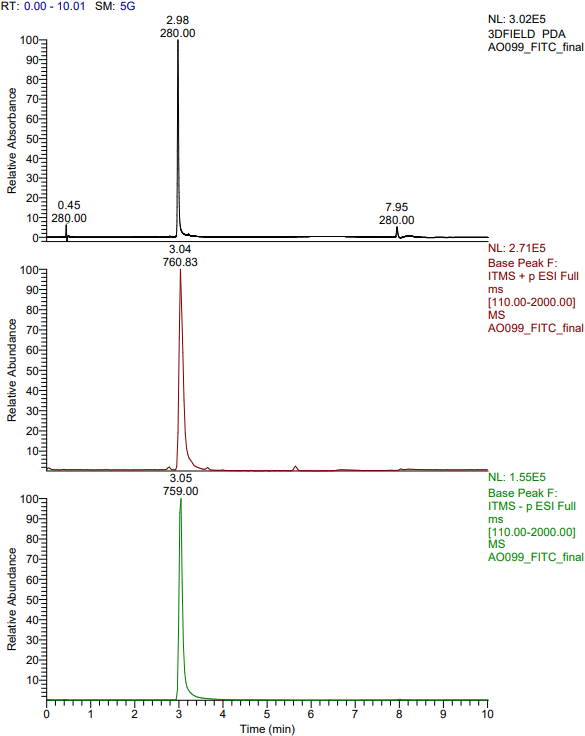


F4

Peptide **F4** was synthesized using GP1 to couple Fmoc-Lys(Alloc)-OH, Fmoc-β-Ala-OH and Fmoc-Lys(Boc)-OH to the Rink amide resin (200 mg, 0.1 mmol), after which GP2 was carried out with 5-hexyn-1-ol (**8**) (6 eq, 0.6 mmol, 42.9 mg). The linear peptide was further extended following GP1. Subsequently, the ring-closing reaction was performed using GP4 and the peptide was deprotected and cleaved from the resin and purified according to GP5. After the purified fractions were collected and lyophilized **F4** (4.2 mg, 2.8%) was obtained as a white amorphous powder. LCMS (ESI): *m/z* 1533 ([M + H]^+^) t_R_ 3.08 min. HRMS (ESI): m/z = 767.2870 calculated for [C70H85N16O20PS + 2H]^2+^; found: 767.2875.

**
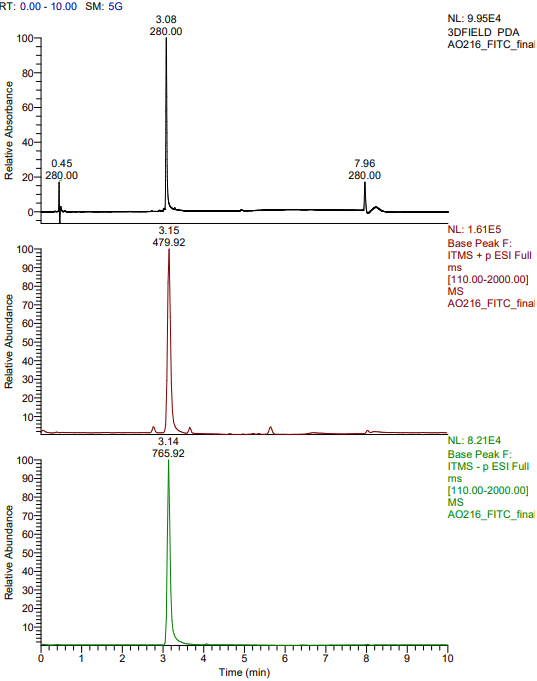
**

2

Peptide **2** was synthesized using GP1 to couple Fmoc-Lys(Alloc)-OH, Fmoc-β-Ala-OH and Fmoc-Lys(Boc)-OH to the Rink amide resin (200 mg, 0.1 mmol) , after which GP2 was carried out with 3-butyn-1-ol (**6**) (6 eq, 0.6 mmol, 35.7 mg). The remaining amino acids were added following GP1. Subsequently, the ring-closing reaction was performed using GP4 before removal of the Alloc protecting group and acetylation of the free Lys sidechain and the peptide was deprotected and cleaved from the resin and purified according to GP5. After the purified fractions were collected and lyophilized, **2** (0.6 mg, 0.5%) was obtained as a white amorphous powder. LCMS (ESI): *m/z* 1157 ([M + H]^+^) t_R_ 2.39 min.


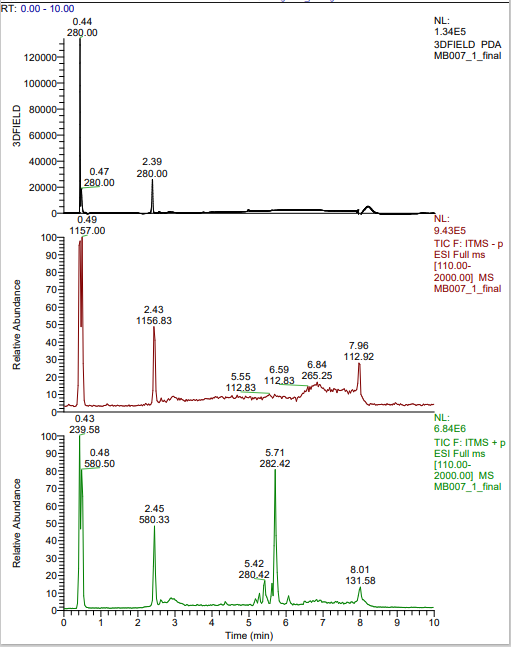


3

Peptide **3** was synthesized using GP1 to couple Fmoc-Lys(Boc)-OH to the Rink amide resin (200 mg, 0.1 mmol) , after which GP2 was carried out with 4-pentyn-1-ol (**7**) (6 eq, 0.6 mmol, 42.9 mg). The linear peptide was further extended following GP1. Subsequently, the ring-closing reaction was performed using GP4 and the peptide was deprotected and cleaved from the resin and purified according to GP5. After the purified fractions were collected and lyophilized, **3** (3.0 mg, 3.3%) was obtained as a white amorphous powder. LCMS (ESI): *m/z* 931 ([M + H]^+^) t_R_ 2.28 min. HRMS (ESI): m/z = 466.1953 calculated for [C39H55N12O13PS + 2H]^2+^; found: 466.1955.


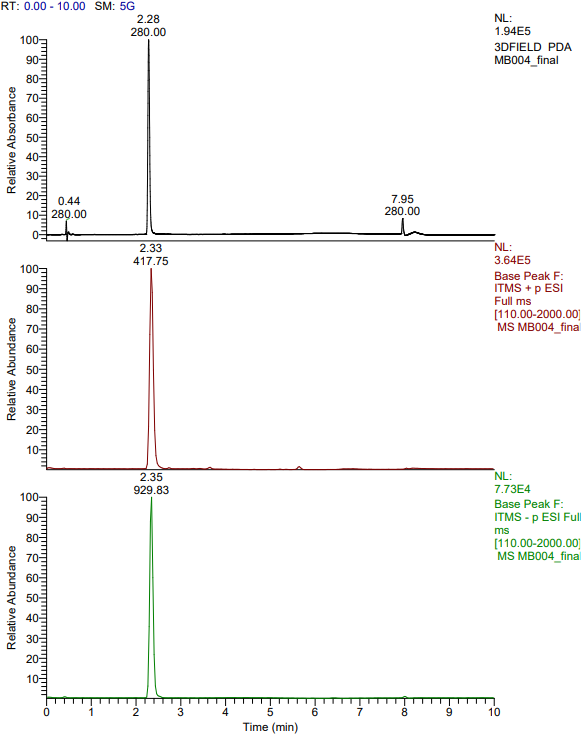


4

Peptide **4** was synthesized using GP1 to couple Fmoc-Lys(Boc)-OH to the Rink amide resin (200 mg, 0.1 mmol) , after which GP2 was carried out with 5-hexyn-1-ol (**8**) (6 eq, 0.6 mmol, 33.6 mg). The linear peptide was further extended following GP1. Subsequently, the ring-closing reaction was performed using and the peptide was deprotected and cleaved from the resin and purified according to GP5. After the purified fractions were collected and lyophilized, **4** (5.0 mg, 5.2%) was obtained as a white amorphous powder. LCMS (ESI): *m/z* 944 ([M + H]^+^) t_R_ 2.53 min. HRMS (ESI): m/z = 473.2031 calculated for [C40H57N12O13PS + 2H]^2+^; found: 473.2028.


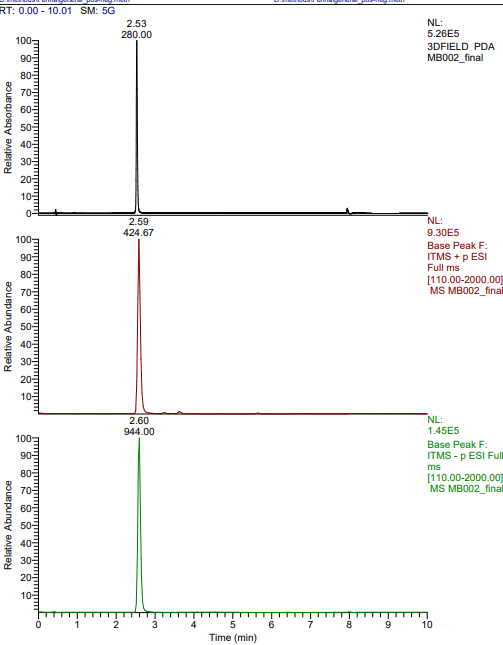


Supporting references

[1] G. Winter, D. G. Waterman, J. M. Parkhurst, A. S. Brewster, R. J. Gildea, M. Gerstel, L. Fuentes-Montero, M. Vollmar, T. Michels-Clark, I. D. Young, N. K. Sauter, G. Evans. DIALS: Implementation and Evaluation of a New Integration Package. *Acta Crystallogr D Struct Biol* **2018**, *74*(Pt 2), 85–97.

[2] A. Vagin, A. Teplyakov. Molecular Replacement with MOLREP. *Acta Crystallogr D Biol Crystallogr* **2010**, *66*(Pt 1), 22–25.

[3] G. N. Murshudov, P. Skubák, A. A. Lebedev, N. S. Pannu, R. A. Steiner, R. A. Nicholls, M. D. Winn, F. Long, A. A. Vagin. REFMAC5 for the Refinement of Macromolecular Crystal Structures. *Acta Cryst D* **2011**, *67*(4), 355–367.

[4] P. Emsley, K. Cowtan. Coot: Model-Building Tools for Molecular Graphics. *Acta Crystallogr D Biol Crystallogr* **2004**, *60*(Pt 12 Pt 1), 2126–2132.

[5] Deposition numbers 9SA9 (for **2**), 9SAA (for **3**), 9SAB (for **4**), 9SAC (for ChREBP and **3**), and 9SAE (for ChREBP and **4**) contain the supplementary crystallographic data for this paper.
